# The cloacal outgrowth orchestrates co-development of the bladder and umbilical arteries

**DOI:** 10.1242/dev.205398

**Published:** 2026-08-05

**Authors:** Xing Ye, Liguang Xia, Xianfa Yang, Ruirong Tan, Haochuan Zhang, Liming Lei, Jungang Huang, Yingsheng Zhang, Edward Morrisey, Ping Zhu, Zhongrong Li, Naihe Jing, Xue Li

**Affiliations:** ^1^ Department of Medicine, Department of Biomedical Sciences, Samuel Oschin Comprehensive Cancer Institute, Cedars-Sinai Medical Center, Cedars-Sinai Health Sciences University, 8700 Beverly Blvd, Los Angeles, CA 90048, USA; ^2^ Department of Urology, Boston Children's Hospital, Harvard Medical School, 300 Longwood Ave, Boston, MA 02115, USA; ^3^ Department of Pediatric Surgery, The Second Affiliated Hospital and Yuying Children's Hospital of Wenzhou Medical University, Wenzhou 325027, Zhejiang Province, People's Republic of China; ^4^ Guangdong Provincial Key Laboratory of Stem Cell and Regenerative Medicine, Guangzhou Institutes of Biomedicine and Health, Chinese Academy of Sciences, Guangzhou 510005, People's Republic of China; ^5^ Guangdong Cardiovascular Institute, Guangdong Provincial People's Hospital, Guangdong Academy of Medical Sciences, Southern Medical University, Guangzhou, Guangdong 510100, People's Republic of China; ^6^ Department of Medicine, Department of Cell and Developmental Biology, University of Pennsylvania, Philadelphia, PA 19104, USA; ^7^ Institute for Stem Cell and Regeneration, Chinese Academy of Sciences, Beijing 100101, People's Republic of China

**Keywords:** *Shh*, *Wnt2*, Geo-seq, High resolution episcopic microscope (HREM), Cloaca, Allantois, Bladder, Umbilical artery

## Abstract

The urinary bladder is an innovation of eutherian mammals to manage liquid waste. However, bladder development and its embryonic function remain poorly understood. Here, we report that in both humans and mice the bladder emerges from the cloaca as a rostral outgrowth, which is flanked by the umbilical arteries. Arrest of the outgrowth, as seen in *Shh-*null mouse embryos, causes bladder agenesis and unexpected severe defects of the umbilical arteries, suggesting that the outgrowth epithelial signal *Shh* coordinates development of both the bladder and the umbilical arteries. The signaling molecule *Wnt2* is restricted to the rostral outgrowth mesenchyme and its expression is dramatically downregulated in *Shh-*null mutants. Furthermore, *Wnt2-*null mutants develop a small bladder and single umbilical artery defects. Collectively, we uncover an unexpected co-development mechanism of the bladder and umbilical arteries orchestrated by the cloacal outgrowth signals. The co-development paradigm offers a conceptual framework to understand evolution and development of the bladder.

## Teaser

**Summary:** 3D anatomical analysis in mouse and human demonstrates co-development of the bladder and the umbilical arteries, orchestrated by the Shh-Wnt2 genetic pathway, highlighting that, besides managing metabolic waste, the bladder is required for survival of mammalian embryos.

## INTRODUCTION

The urinary bladder is one of the evolutionary innovations of amniotes: it is found in placental mammals but is largely absent from reptiles and birds (Hicks, 1975; Bentley, 1979). Having the bladder offers selective advantages in water preservation (Hicks, 1975) and predator evasion (McCarthy and McCarthy, 2019) for terrestrial vertebrates to thrive on dry land. While the bladder is known for managing liquid metabolic waste after birth, formation and function of the bladder before birth are often overlooked and poorly understood.

The role of embryonic bladder, particularly at the stages prior to renal function, remains largely unknown. The mouse bladder emerges several days before the kidneys begin to produce urine (Rasouly and Lu, 2013; Georgas et al., 2015; Huang et al., 2016; Kruepunga et al., 2018; de Bakker et al., 2016). Similarly, the human bladder also emerges prior to renal function, implying that the embryonic bladder is unlikely to play a role in waste management. Indeed, mammalian embryos dispose of their metabolic waste primarily through the umbilical arteries to the chorioallantoic placenta or placenta, where liquid metabolic waste is removed maternally.

In humans, congenital abnormalities involving isolated bladder agenesis or absence of the bladder are rare with an estimated incidence of 1:600,000 (Glenn, 1959; Yahya, 2023). The majority of affected individuals exhibit varying degrees of genital abnormalities, from ambiguous to normal genitalia. Unexpectedly, umbilical artery defects are also observed among individuals with bladder agenesis, including the single umbilical artery phenotype, absence of a common iliac artery, and iliac artery anomaly (Dykes et al., 1993; Lowrey et al., 2019). Absence of the bladder is often found in sirenomelia (1.5-4.2 cases per 100,000) and caudal dysgenesis (1-2.5 cases per 100,000) (Thottungal et al., 2010; Garrido-Allepuz et al., 2011). This is consistent with the notion that the cloaca functions as an organizing center to instruct development of caudal structures, including the bladder, genital tubercle, and the hindlimbs (Tschopp et al., 2014; Herrera and Cohn, 2014; Lozovska et al., 2024). Bladder agenesis is also observed in rodents when pregnant females are exposed to Adriamycin (doxorubicin), a chemotherapy medication (Thompson et al., 1978). In this teratogenesis model, the cranial portion of the bladder to the point of entries of Wolffian ducts (WDs) is absent (Liu et al., 1999), suggesting that this region, constituting the main body of the urinary bladder, originates from a unique population of progenitor cells that are particularly sensitive to the teratogen exposure. Together, the bladder is an integral component of the caudal structures, which also include the genital tubercle, hindlimb, and possibly the umbilical arteries; furthermore, the cloaca is central to normal development of the bladder and is required for mammalian embryos to thrive inside the womb.

The cloaca is a caudal extension and local expansion of the hindgut, which is formed around embryonic day (E) 10.5 in mice and the fourth week of gestation in humans (Matsumaru et al., 2015; Huang et al., 2016; Kruepunga et al., 2018; Liaw et al., 2018). For reptiles and birds, which reproduce inside cleidoic eggs, embryonic waste drains from the cloaca to the allantois, an extra-embryonic sac functioning like an embryonic bladder. The cloaca persists after hatching and the adult cloaca serves as an opening shared by the digestive, urinary and reproductive tracts of reptiles and birds. The cloaca–allantois conduit is conserved among vertebrates. However, unlike the cloaca from reptiles and birds, the mammalian cloaca undergoes dramatic morphogenetic changes, in part due to the region-specific recruitment and asymmetric growth of peri-cloacal mesenchyme (PCM), such as intra-cloacal mesenchyme or the urorectal septum as well as the dorsal PCM (Wang et al., 2011, 2013; Guo et al., 2014). This morphologic transformation divides the cloaca into two halves: the ventral half or urogenital sinus (UGS) differentiates into the lower urinary tract while the dorsal half contributes to the anorectal canal of the digestive tract (Matsumaru et al., 2015; Huang et al., 2016; Kruepunga et al., 2018). Consequently, the urinary and digestive tracts are connected in reptiles and birds but separated in mammals. For reasons that are largely unknown, the bladder emerges at the junction between the UGS and the allantois in placental mammals (Mossman, 1987; Bentley, 1979; van der Putte, 2006).

In a mouse line deficient of transmembrane protein 132a (*Tmem132a^−/−^*) (Zeng and Liu, 2023), the gut fails to extend caudally to form the cloaca at E9.5. Consequently, the bladder as well as the genital tubercle are absent from *Tmem132a^−/−^* embryos. Sonic hedgehog (*Shh*) encodes a signaling molecule that is expressed in the cloacal epithelium (Haraguchi et al., 2001, 2007; Perriton et al., 2002). The cloaca is formed initially in *Shh^−/−^* embryos, but the PCM is underdeveloped (Huang et al., 2016), resulting in persistent cloaca and hypoplastic bladder and genital tubercle (Haraguchi et al., 2001, 2007; Perriton et al., 2002). Compared to *Shh^−/−^* mutation alone, compound mutants deficient of both *Shh* and bone morphogenetic protein *7* (*Bmp7*) exhibit an even more severe phenotype in which the genital tubercle is absent and the hindlimbs are fused (Garrido-Allepuz et al., 2012), resembling the human congenital abnormality sirenomelia. The cloaca of *Shh;Bmp7* compound mutants is narrower than normal and shows a reduced local expansion at E9.5. The sirenomelia-like phenotype is also observed in two other mouse models, including a compound mouse mutant line lacking both *Bmp7* and twisted gastrulation (*Tsg*; *Twsg1*) (Zakin et al., 2005) and a mouse line with conditional knockout of *Bmp4* (Suzuki et al., 2012). Therefore, formation of the cloaca is the crucial first step towards formation of the bladder. How the bladder emerges from the cloaca at the junction between the UGS and allantois remains largely unknown.

Here, we investigate bladder development in humans and mice. Our study uncovers an unexpected co-development mechanism of the bladder and umbilical arteries, which is controlled in part by epithelial and mesenchymal signals of the cloacal outgrowth. The co-development paradigm offers a conceptual framework to understand the development and evolution of mammalian caudal structures as well as human congenital abnormalities such as bladder agenesis and sirenomelia.

## RESULTS

### Mammalian bladder originates from the cloaca instead of the allantois

**Fig. 1.**
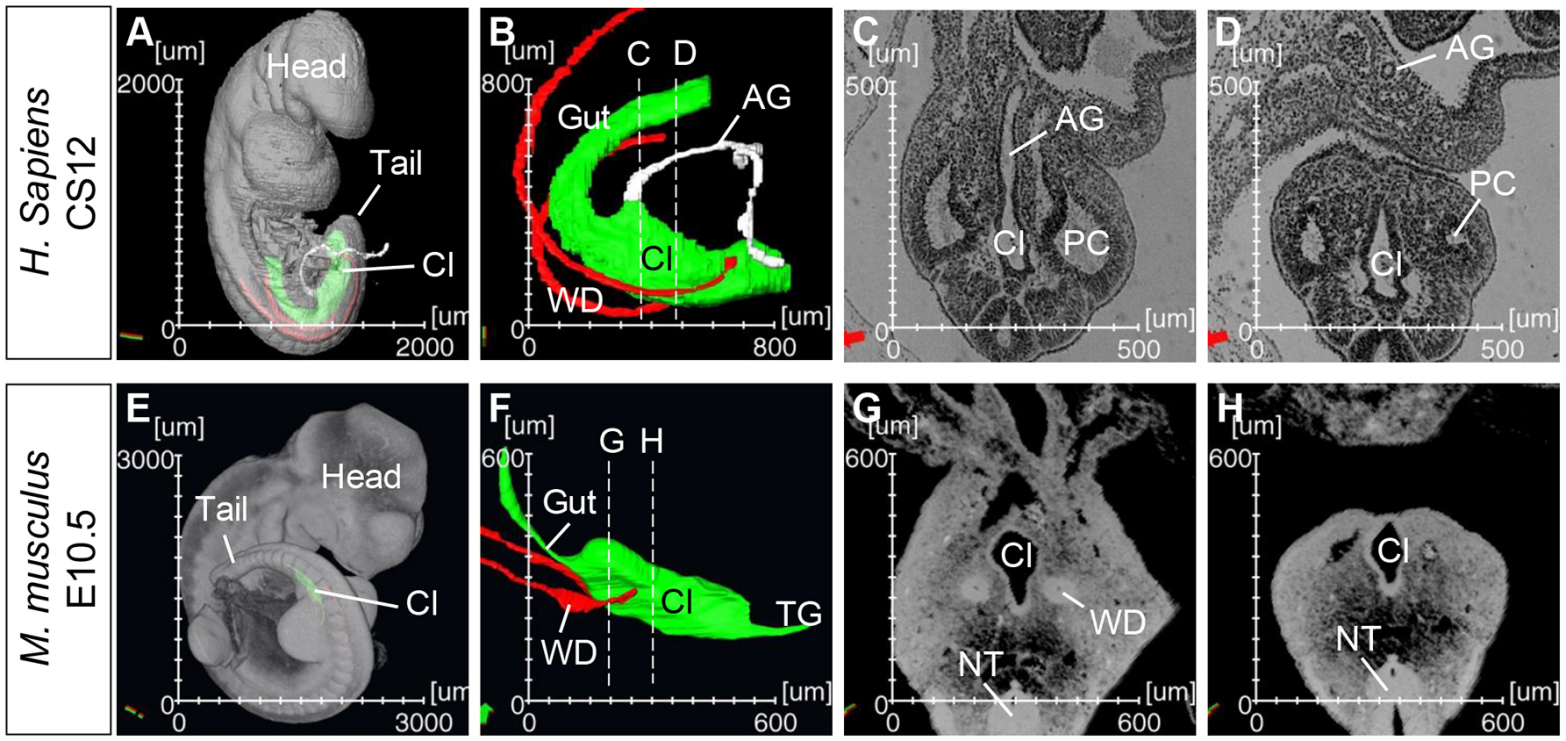
**Species variations of the allantoic gut.** (A-H) The allantoic gut is found in human (A-D) but not mouse (E-H) allantois. (A,E), Whole-mount ventrolateral views of CS12 human (A; *n*=1) and E10.5 mouse (E; *n*=5) embryos. (B,F) 3D overview of the cloaca and related structures based on HREM. Green, definitive gut endoderm and cloacal epithelium; white, allantoic gut; red, Wolffian duct (WD). (C,D,G,H) Virtual digital cross-sections of the cloaca taken from the planes shown in B and F (dashed lines). AG, allantoic gut; Cl, cloaca; NT, neural tube; PC, peritoneal cavity; TG, tail gut.

It has been postulated that the urinary bladder originates from both the cloaca and the allantois, with the neck or proximal part of the urinary bladder deriving from the cloaca and the remainder of the bladder, such as the bladder dome, from the allantois (Bentley, 1979; van der Putte, 2006). To test this hypothesis directly, we tracked the developmental trajectory of the allantois of human and mouse embryos using a comprehensive series of digitized high-resolution episcopic microscope (HREM) 3D images (Tables S1 and S2) (de Bakker et al., 2016; Huang et al., 2016; Kruepunga et al., 2018). In human embryos, formation of the cloaca-allantois canal was observed initially at Carnegie stage (CS) 8 as an endoderm protrusion that extended into the body stalk (Fig. S1A-D, Movie 1). The canal, which connects the cloaca directly to the allantois to form a continuous embryonic–extra-embryonic conduit, was clearly visible at CS12 (Fig. 1A-D). Unlike observations in bird and reptile embryos (Mossman, 1987), however, the human allantoic gut was only a rudimentary structure and became progressively smaller at later stages (CS14, 18 and 21; Movie 2).

Surprisingly, the cloaca-allantois canal was not observed in mouse embryos at the comparable stages (Fig. 1E-H, Movie 3). Instead, mouse cloaca ended as a closed-off structure at the caudal end of the trunk. To rule out the possibility that the presumptive allantoic gut might have formed initially, but was degenerated thereafter, we examined E8.5 mouse embryos, a stage comparable to CS8 human embryos. Unlike human embryos, an expected endodermal protrusion was never observed in mouse embryos based on the HREM images (Fig. S1E-H). To ascertain this finding, we analyzed serial histological sections, which showed that the hindgut was formed, but ended as a closed-off structure ventral to the caudal neuropore of E8.5 mouse embryos (Fig. S1I-K). In comparison, the allantois at this stage (E8.5) had already established an elaborate extra-embryonic vascular network with no evidence of the cloaca-allantois canal. Absence of the cloaca-allantois canal in mouse embryos raises a strong possibility that the urinary bladder originates from the cloaca.

### The bladder emerges as the rostral outgrowth of the cloaca

**Fig. 2.**
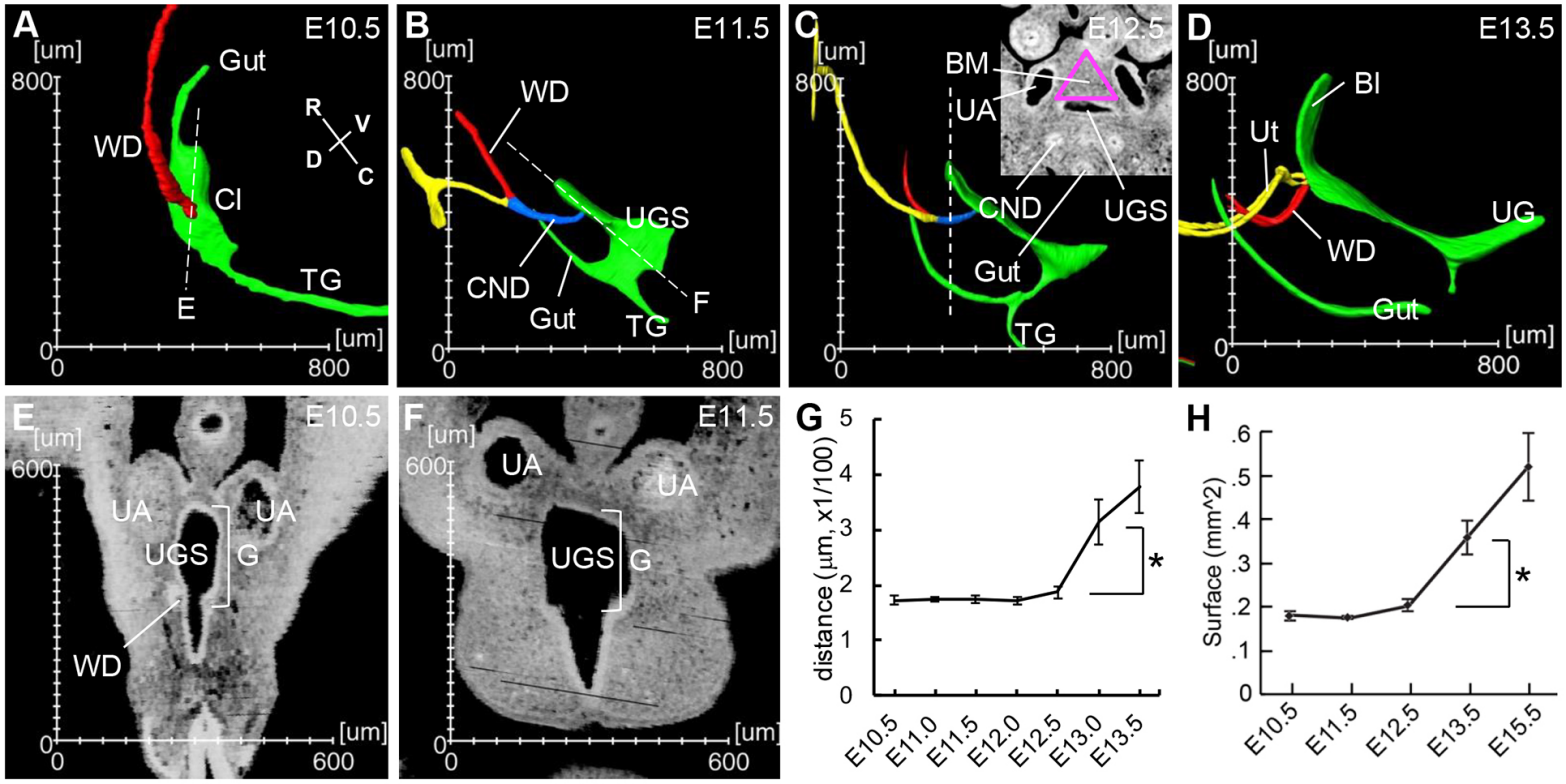
**Rostral outgrowth of the cloaca forms the bladder.** (A-D) 3D sagittal views of mouse cloaca from E10.5 to E13.5. Inset in C shows a digital cross-section from the plane shown as dashed lines. The triangular mesenchymal domain is marked in magenta. (E,F) Digital sections through the planes shown in A and B, respectively. Green, definitive gut endoderm and cloacal epithelium; white, allantoic gut; red, Wolffian duct; yellow, ureter; blue, common nephric duct; magenta, bladder mesenchyme. (G) Distance from insertion site of the Wolffian duct to the rostral tip of the cloaca as shown by brackets in E and F. (H) Surface area of the cloaca. *P<0.05, compared with E12.5 (unpaired, one-tailed Student's *t-*test). *n*=3 (E10.5, 11.5, 12.0, 12.5, 13.0 and E13.5), *n*=2 (E11.0). Rostral (R)-caudal (C) and dorso (D)-ventral (V) axes are indicated. Bl, Bladder; BM, bladder mesenchyme; Cl, cloaca; CND, common nephric duct; TG, tail gut; UA, umbilical artery; UGS, urogenital sinus: Ut, ureter; WD, Wolffian duct.

To further examine this possibility, we investigated how the bladder emerges from the cloaca in human and mouse embryos. First, we used the WD insertion sites as spatial references because they are considered to be stable anatomical landmarks along the rostrocaudal axis of the cloaca (Kruepunga et al., 2018). In mouse embryos, the WDs inserted into the cloaca at E10.5 (Fig. 2A). At E11.5 and E12.5, the UGS constituted the ventral half of the cloaca oriented along the rostro-caudal axis (Fig. 2B-D). At E12.5, rostral outgrowth of the cloaca was observed, which inserted into the triangular mesenchymal space (Fig. 2C, inset). By E13.5, the rostral outgrowth became a prominent hollow structure, representing appearance of the bladder as a distinct anatomical structure (Fig. 2D). Concomitant to formation of the bladder at the rostral end of the cloaca, a caudal extension of the cloaca was also observed, resulting in formation of the genital tubercle (Fig. 2C,D).

These observations suggest that the bladder emerges from the rostral outgrowth of the cloaca. To substantiate this conclusion, we measured the distance from the WD insertion sites to the tip of rostral outgrowth and the epithelial surface area of the entire cloaca (Fig. 2E-H). The distance remained constant from E10.5 (∼172.67 μm) to E12.5 (∼187.33 μm) but increased significantly from E12.5 (187.33 μm) to E13.0 (∼314.67 μm) and to E13.5 (∼378.33 μm) (Fig. 2G; *n*=3, *P*<0.05, unpaired, one-tailed Student's *t-*test). The overall surface area of the cloaca was unchanged from E10.5 to E12.5 but began to increase significantly at E12.5 (Fig. 2H; *n*=3, *P*<0.05, unpaired, one-tailed Student's *t-*test). These findings suggest that transformation of the cloaca precedes the outgrowth, and further confirm that the outgrowth of the cloaca precedes formation of the bladder rostrally and the genital tubercle caudally.

**Fig. 3.**
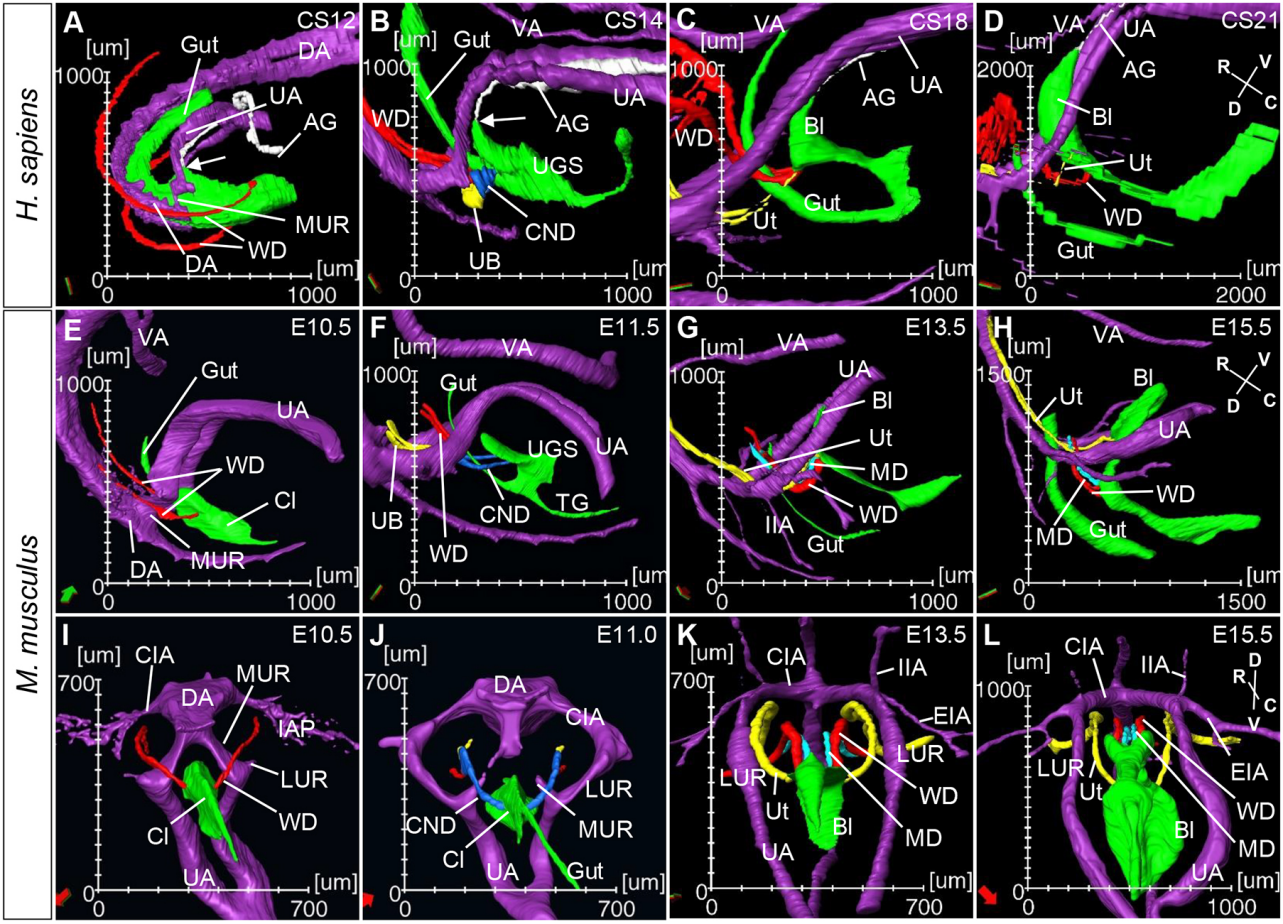
**Spatiotemporal correlation of the rostral outgrowth of the cloaca and remodeling of the umbilical arteries in human and mouse**. (A-H) 3D sagittal views of outgrowth of the cloaca and remodeling of the umbilical arteries in human (A-D) and mouse (E-H) embryos at comparable developmental stages. (I-L) Caudal views of mouse umbilical arteries reveal the critical periods of umbilical artery remodeling between E10 and E11, and maturation of the iliac arteries between E13 and E15 in mice. Green, definitive gut endoderm and cloacal epithelium; white, allantoic gut; red, Wolffian duct; yellow, ureter; blue, common nephric duct; purple, arterial vessels; cyan, Mullerian duct. Arrows indicate the junction between the cloaca and the allantois. Rostral (R)-caudal (C) and dorso (D)-ventral (V) axes are indicated. AG, Allantoic gut; Bl, bladder; Cl, cloaca; CIA, common iliac artery; CND, common nephric duct; DA, dorsal aorta; EIA, external iliac artery; IAP, iliac artery plexus; IIA, internal iliac artery; LUR, lateral umbilical artery root; MD, Mullerian duct; MUR, medial umbilical artery root; TG, tail gut; UA, umbilical artery; UB, ureteric bud; UGS, urogenital sinus; Ut, ureter; VA, vitelline artery; WD, Wolffian duct.

The umbilical arteries have been used as another independent anatomical landmark along the rostrocaudal axis (Kruepunga et al., 2018; Hikspoors et al., 2015). The human cloaca-allantois canal traversed between the umbilical arteries (Fig. 3A-D). The junction between the cloaca and allantois, marked by the transition from pseudostratified columnar epithelium to low cuboidal epithelium, respectively (Kruepunga et al., 2018), was located near the root of umbilical arteries (Fig. 3A-D, arrows). A rostral expansion of the cloaca was visible at CS18 and progressed to form the bladder flanked by the umbilical arteries (Fig. 3C,D). In mouse embryos, the rostral outgrowth was also flanked by the umbilical arteries at the comparable developmental stages (Fig. 3E-H). The rostral tip of the cloaca was located near the umbilical arteries at E10.5 and E11.5. By E13.5 and E15.5, the bladder was formed between the umbilical arteries. Together, these findings support the notion that the bladder emerges from the rostral outgrowth of the cloaca in humans and mice.

### The rostral outgrowth coincides with remodeling of the umbilical arteries

The umbilical arteries are the vital structures that carry deoxygenated blood and metabolic waste from the fetus to the placenta. The umbilical and fetal circulatory systems form independently. In mice, the umbilical artery originates from mesoderm-derived allantoic angioblasts and first appears around E8.5. At approximately the same time, an independent vessel of confluence emerges at the base of the allantois closest to the embryo (Downs and Rodriguez, 2020). It has been proposed that the vessel of confluence differentiates into the umbilical artery root that connects directly to the embryonic dorsal aorta. However, remodeling of the umbilical artery roots, particularly with respect to their potential relationship to bladder development, has not been reported to date.

Physical proximity between the developing bladder and umbilical arteries prompted us to examine whether these two seemingly unrelated structures are developmentally linked. During the crucial period of bladder development, the medial umbilical artery roots (MURs) were connected directly to the descending aorta at E10.5 in mice and CS12 in humans (Fig. 3A,E,I). At this stage, the WDs traversed laterally to the MURs. Surprisingly, the spatial relationship between the WDs and umbilical arteries was reversed at later stages (after E11.5), with the WDs traversing medially instead (Fig. 3B-D,F-H), indicating a dramatic remodeling of the umbilical arteries.

Possible remodeling of the umbilical arteries became self-evident when embryos were visualized caudally (Fig. 3I-L). The MURs were prominent at E10.5 but had degenerated half a day later at E11.0 (compare Fig. 3I and J). The lateral umbilical artery roots (LURs) emerged from the umbilical arteries and the iliac artery plexus appeared from the descending aorta at E10.5 (Fig. 3I). Concomitant with degeneration of the MURs at E11.0, the mature LURs were formed, which connect the descending aorta indirectly via the common iliac arteries (Fig. 3K,L). As a result, the umbilical arteries traversed laterally to the WDs, marking completion of the umbilical artery remodeling, i.e. degeneration of the MURs and emergence of the LURs and common iliac arteries. Although the mechanism of the umbilical artery remodeling remains to be elucidated, the process of remodeling appears to be conserved in humans and mice.

### *Shh* orchestrates co-development of the bladder and umbilical arteries

To test the hypothesis that the bladder and umbilical arteries are mechanistically linked during embryonic development, we focused on signaling molecule *Shh*, which is restrictively expressed in the cloacal epithelium and absent from the cloacal mesenchyme (Seifert et al., 2008). The cloaca was formed in *Shh* null-mutant (*Shh^−/−^*) embryos at E10.5 (Movie 4). 3D HREM analyses of *Shh^−/−^* embryos also revealed that septation of the cloaca was initiated but the process failed to complete in the mutants, resulting in a persistent cloaca-like phenotype. In addition, the rostral and caudal outgrowth of the UGS was arrested at E12.5, resulting in agenesis of the urinary bladder and genital tubercle as reported previously (Haraguchi et al., 2001, 2007; Perriton et al., 2002). Stage-specific deletions of *Shh* further indicate an early phase requirement of *Shh* signaling cloacal septation, formation of the bladder and genital tubercle, and a later phase requirement of external genital development (Seifert et al., 2009; Miyagawa et al., 2009; Lin et al., 2009). Together, these findings show that epithelial *Shh* signal is required for formation of the bladder and genital tubercle.

The role of hedgehog signaling in vascular development, such as remodeling of the pharyngeal arch arteries and the fetal–placental arterial connection, has also been implicated previously (Downs and Rodriguez, 2020; Chapouly et al., 2019; Kolesova et al., 2008). However, a possible role for *Shh* in remodeling of the umbilical artery roots has not been reported. To ascertain that *Shh* is absent from any progenitor cells that may contribute directly to the umbilical arteries, we used the Cre/loxP genetic strategy to indelibly label *Shh-*expressing cells as well as their progenies, i.e. the *Shh* genetic lineage (Harfe et al., 2004). The labeled cells were visualized as whole mounts using 3D HREM. Strong signals were detected in all tissues that are known to originate from the *Shh-*expressing cells, including the gut epithelium, cloacal epithelium, notochord, floorplate, and the zone of polarized activity (Fig. S2, Movie 5). Notably, no umbilical structures were labeled, indicating that they do not belong to the *Shh* genetic lineage. Therefore, the *Shh-*expressing progenitors do not contribute directly to the umbilical arteries.

**Fig. 4.**
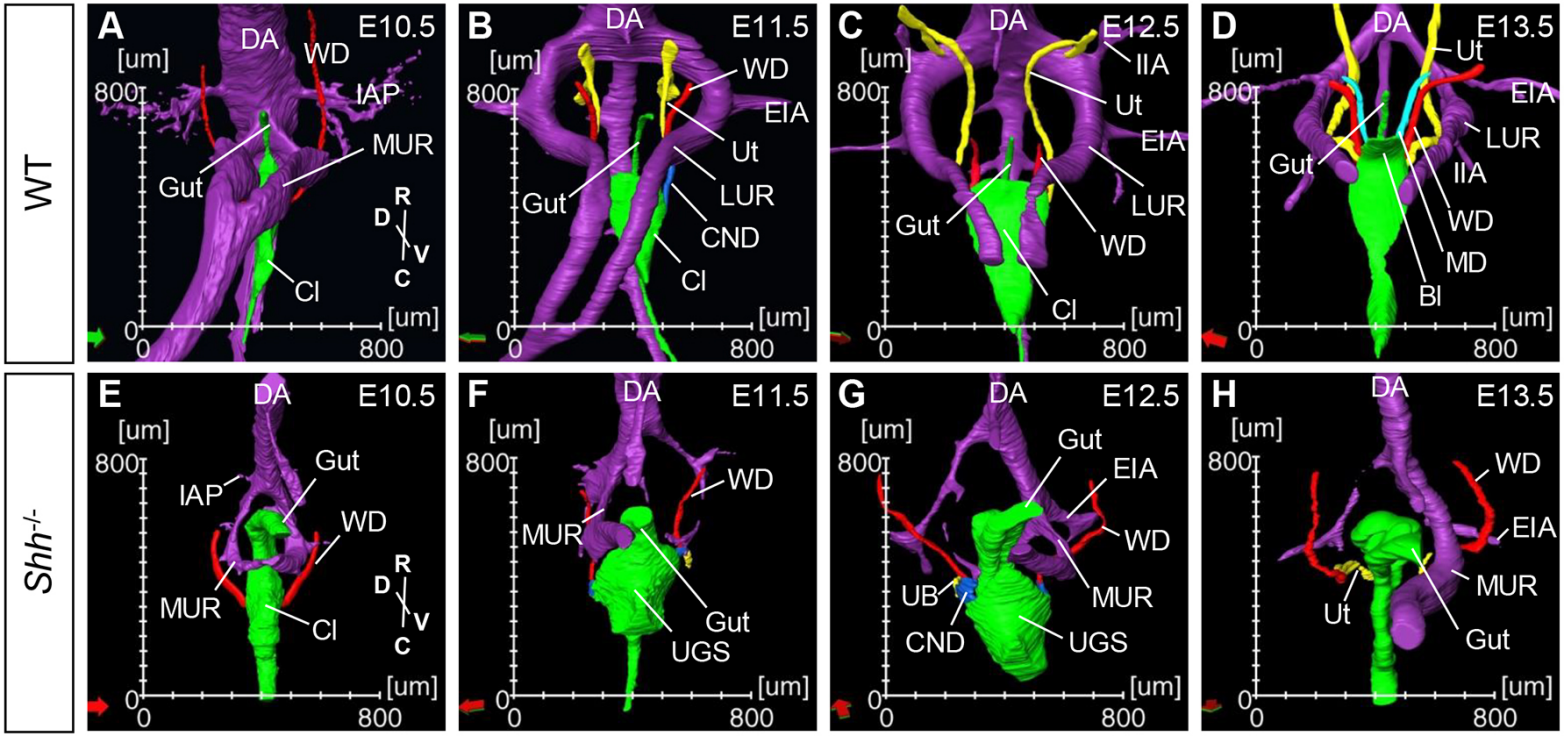
***Shh* is required for remodeling of the umbilical arteries.** (A-H) 3D ventral views of the arterial networks (purple), the cloaca/gut epithelium (green) and associated structures of wild-type (WT; A-D) and *Shh^−/−^* mutant (E-H) embryos from E10.5 to E13.5 (*n*=3 for each stage). Apparent umbilical artery defects provide evidence that the medial umbilical arteries are lost at one side, and the lateral umbilical arteries do not form (F-H). Green, definitive gut endoderm and cloacal epithelium; white, allantoic gut; red, Wolffian duct; yellow, ureter; blue, common nephric duct; purple, arterial vessels; cyan, Mullerian duct. Rostral (R)-caudal (C) and dorso (D)-ventral (V) axes are indicated. Bl, bladder; Cl, cloaca; CND, common nephric duct; DA, dorsal aorta; EIA, external iliac artery; IAP, iliac artery plexus; IIA, internal iliac artery; LUR, lateral umbilical artery root; MD, Mullerian duct; MUR, medial umbilical artery root; UB, ureteric bud; UGS, urogenital sinus; Ut, ureter; WD, Wolffian duct.

We next examined whether *Shh* may indirectly affect development of the umbilical arteries in a paracrine fashion. We analyzed a series of *Shh^−/−^* embryos from E10.5 to E13.5 (Fig. 4, Movie 4). The vascular network including the umbilical arteries and dorsal aorta was highlighted to reveal vascular anomalies of *Shh^−/−^* mutants using wild-type littermates as controls. The umbilical arteries were observed in *Shh^−/−^* mutants; however, these arteries at E10.5 were apparently smaller in size compared to controls (Fig. 4A,E). The iliac artery plexus was clearly underdeveloped at this stage. Moreover, it failed to progress to form the LURs at later stages between E11.5 and E13.5 (Fig. 4B-D,F-H). Unlike wild-type controls, in which the MURs were degenerated at E11.5, the MURs persisted in *Shh^−/−^* mutants, resulting in the single umbilical artery phenotype at E13.5 (Fig. 4, compare B-D to F-H). The single artery traversed medially to the WD, consistent with the possibility that it originated from the MUR instead of LUR arteries (Fig. 4H). Collectively, these findings suggest that Shh is a paracrine epithelial signal orchestrating co-development of the bladder and umbilical arteries.

### *Wnt2* is an outgrowth mesenchymal signal required for co-development of the bladder and the umbilical arteries

**Fig. 5.**
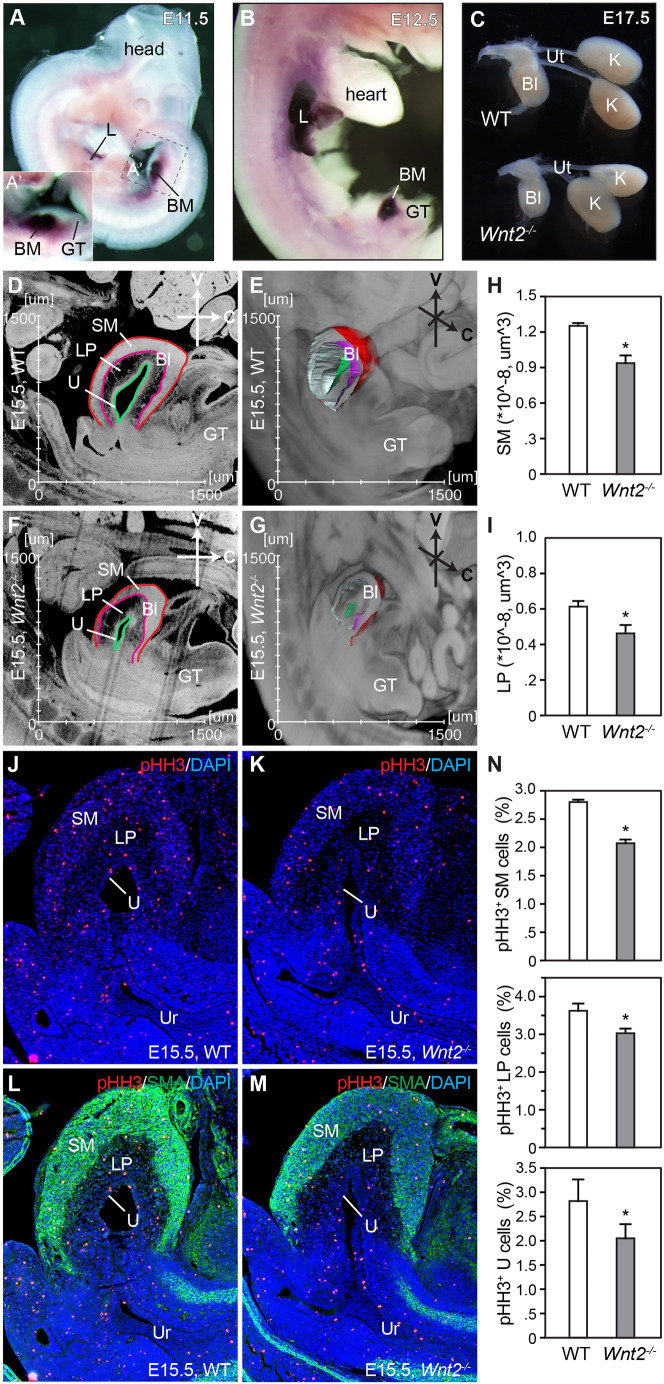
***Wnt2* regulates growth of the bladder.** (A,B) *Wnt2* is restrictedly expressed in the developing bladder and lung. Whole-mount RNA *in situ* hybridization of mouse embryos at E11.5 (A) and E12.5 (B) using a *Wnt2-*specific probe (dark purple staining). (C) *Wnt2-*deficient (*Wnt2^−/−^*) bladders are smaller than those of wild-type (WT) controls at E17.5. Whole-mount overview of the kidneys and bladder. (D-G) Representative HREM 2D (D,F) and 3D (E,G) images of the bladder (midline sagittal cut). Green, bladder lumen; magenta, boundary of the laminar propria and smooth muscle layers of the bladder; red, outline of the bladder. Ventral and caudal axes are indicated. (H,I) Volumetric quantifications of the smooth muscle layer (H) and the lamina propria layer (I) of bladders at E15.5 (before urine production). (J-M) Immunofluorescence staining of E15.5 WT and *Wnt2* mutant (*Wnt2^−/−^*) embryos using phospho-histone H3 (pHH3) and smooth muscle actin (SMA) antibodies. (N) Quantification of pHH3-positive cells as shown in J,K. *P<0.05 (*n*=4; unpaired, two-tailed Student's *t-*test). Bl, bladder; BM, bladder mesenchyme; GT, genital tubercle; K, kidney; L, lung; LP, laminar propria; U, urothelium; Ur, urethra; SM, smooth muscle.

**Fig. 6.**
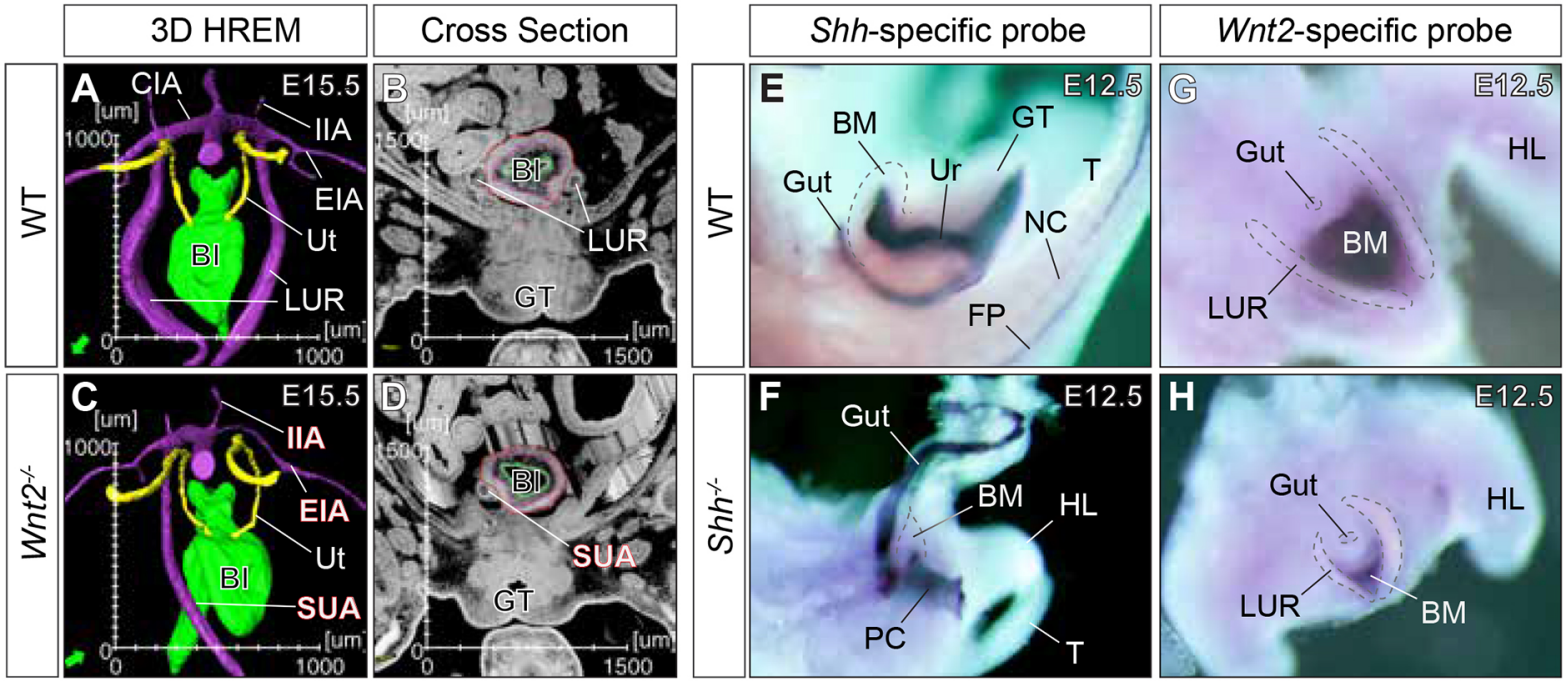
**The *Shh-Wnt2* genetic pathway orchestrates co-development of the bladder and umbilical arteries.** (A-D) HREM visualizations reveal the single umbilical artery and iliac artery defects associated with *Wnt2^−/−^* mutants (C,D). Green, definitive gut endoderm and bladder epithelium; yellow, ureter; purple, arterial vessels. *n*=3. (E-H) *Wnt2* expression is reduced in *Shh^−/−^* mutants. Whole-mount RNA *in situ* hybridization using gene-specific probes as indicated. *n*=3. Bl, bladder; BM, bladder mesenchyme; CIA, common iliac artery; EIA, external iliac artery; FP, floor plate; GT, genital tubercle; HL, hindlimb; IIA, internal iliac artery; LUR, lateral umbilical artery root; NC, notochord; PC, persistent cloaca; SUA, single umbilical artery; T, tail; Ur, urethra; Ut, ureter; WT, wild type.

In addition to the epithelial signal(s), we hypothesized that co-development of the bladder and umbilical arteries may also depend on mesenchymal signal(s). *Wnt2* encodes a canonical Wnt-family signaling molecule and has been independently identified from E16.5 bladder mesenchyme (Jasmine et al., 2025). Previous studies have shown that *Wnt2* is expressed in the foregut mesenchyme and regulates lung development (Goss et al., 2009; Peng et al., 2013). However, its potential role in bladder and umbilical artery development has not been explored. We first validated *Wnt2* expression in the outgrowth mesenchyme from E11.5 to E14.5 using whole-mount RNA *in situ* hybridization (Figs 5, 6, Fig. S2). In addition to the developing lung, strong *Wnt2* signal was detected in the rostral domain of the cloaca at E11.5 and the developing bladder at E12.5 (Fig. 5A,B). The bladder-specific expression pattern became apparent when the stained embryos were examined as histological sections at E13.5 and E14.5 (Fig. S3). A sharp *Wnt2* expression boundary was observed that separated the developing bladder from the developing urethra and genital tubercle (Figs 5B, 6G, Fig. S2B), implying that the bladder mesenchyme is molecularly distinct from the urethral mesenchyme. Together, these findings show that *Wnt2* is highly restricted to the rostral outgrowth mesenchyme, and its expression is maintained in the bladder mesenchyme.

We next examined *Wnt2-*null (*Wnt2^−/−^*) mouse mutants (Goss et al., 2009; Peng et al., 2013). Gross analysis of *Wnt2^−/−^* embryos indicated that the bladder was smaller compared to littermate controls (Fig. 5C), suggesting the function of *Wnt2* is not fully compensated for by *Wnt2b in vivo.* To quantify bladder size, we collected HREM 3D data of E15.5 embryos (Fig. 5D-G), 1 day prior to urine production. Volumetric analysis of both bladder smooth muscle and lamina propria layers showed a significant reduction in *Wnt2^−/−^* mutants compared to controls (Fig. 5H,I). Consistent with this, cell proliferation rates, as indicated by the percentage of phospho-histone H3 (pHH3)-positive cells, were significantly lower in *Wnt2^−/−^* mutants than in controls (Fig. 5J-N). 3D morphometric analysis also revealed that *Wnt2^−/−^* mutants displayed a single umbilical artery phenotype (Fig. 6A-D). Unlike wild-type controls, which had two umbilical arteries traversing bilaterally to the bladder, only one umbilical artery was observed in all *Wnt2^−/−^* mutants (*n*=3). The single umbilical artery was observed either on the left or right side of the bladder. The internal and external iliac arteries were connected aberrantly to the dorsal aorta (Fig. 6C), suggesting that the common iliac artery was also missing in the mutants. Together, these findings demonstrate that *Wnt2* plays a previously unknown role in regulating development of both the bladder and the umbilical arteries.

### Epithelial *Shh* regulates *Wnt2* expression in the bladder mesenchyme

We next examined the possibility whether *Shh* may regulate *Wnt2* gene expression during bladder development. Strong *Shh* expression was detected in the cloacal epithelium as well as the gut epithelium of wild-type embryos (Fig. 6E). The first exon of the *Shh* gene is deleted in *Shh^−/−^* mutants, resulting in a null allele that produces truncated transcripts without any functional protein (Harfe et al., 2004). High levels of truncated *Shh* transcripts were detected in *Shh^−/−^* mutant embryos (Fig. 6F), indicating that *Shh* expression is independent of *Shh* signaling *in vivo*. The expression pattern of the truncated *Shh* transcripts showed that the rostral and caudal outgrowth was absent in *Shh^−/−^* mutant embryos (Fig. 6F), consistent with HREM 3D findings. Strong *Wnt2* transcript signal was observed at the triangular mesenchymal space flanked by the umbilical arteries in wild-type controls. Moreover, *Wnt2* signal was obviously weaker in *Shh^−/−^* embryos (Fig. 6G,H, rostral views), suggesting that epithelial *Shh* signal is required for *Wnt2* expression in the rostral outgrowth mesenchyme during bladder development.

## DISCUSSION

**Fig. 7.**
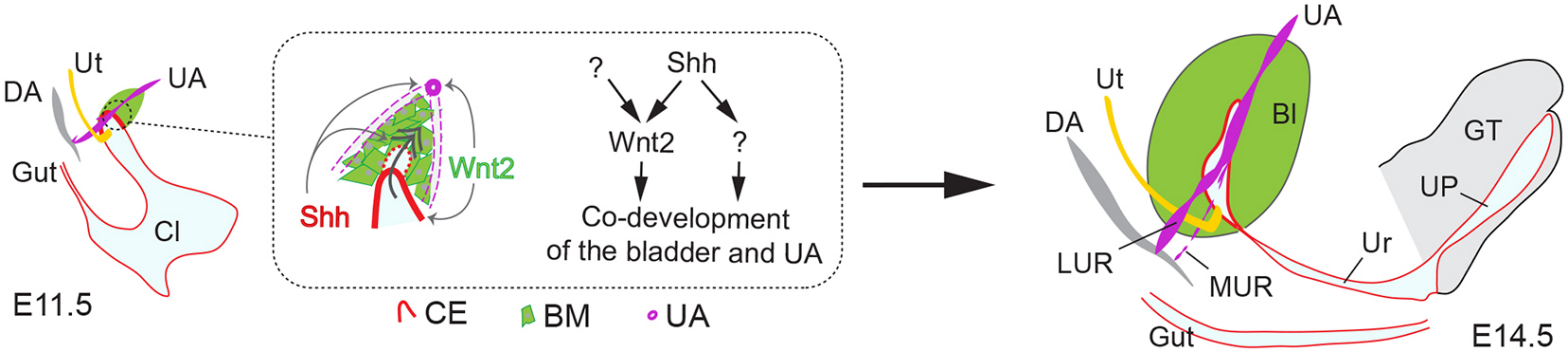
**Proposed model of co-development of the bladder and umbilical arteries.** Rostral outgrowth (double arrow) of the cloaca into the *Wnt2-*positive mesenchymal progenitors (green) results in formation of the bladder. A caudal outgrowth of the cloacal forms the urethra and urethral plate of the genital tubercle. Remodeling of the umbilical artery includes degeneration of the medial umbilical artery roots and establishment of the lateral umbilical roots. *Shh* (red) from cloacal epithelium, which is required for *Wnt2* expression, controls both bladder development and umbilical artery remodeling. *Wnt2* regulates proliferation of bladder mesenchyme and epithelium, as well as remodeling of the umbilical arteries. The co-development program also consists of additional *Wnt2* upstream regulator(s) and *Shh* downstream effector(s). The signals required for rostral outgrowth of the cloaca are unknown but may originate from both the cloacal mesenchyme and the umbilical arteries. Bl, bladder; Cl, cloaca; DA, dorsal aorta; GT, genital tubercle; LUR, lateral umbilical artery root; MUR, medial umbilical artery root; UA, umbilical arteries; UP, urethral plate; Ur, urethra; Ut, ureter.

In this study, we report that the mammalian urinary bladder emerges as a rostral outgrowth of the cloaca (Fig. 7). The outgrowth is mediated in part by the epithelial *Shh* and mesenchymal *Wnt2* signals. Unexpectedly, the outgrowth is required for formation of both the bladder and the umbilical arteries. In addition to supporting life after birth, our findings suggest that having the bladder may also provide a survival advantage before birth by supporting development of the umbilical arteries.

The bladder appears at the junction between the embryonic cloaca and the extra-embryonic allantois. Although we could not rule out a possible contribution of the allantois, especially in humans (Bentley, 1979; van der Putte, 2006), our findings here suggest that the bladder originates from the rostral outgrowth of the cloaca in both humans and mice. For reptiles and birds, the allantoic gut is a vital extra-embryonic organ that stores metabolic waste before hatching. The allantois is conserved in mammals; however, the human allantoic gut is underdeveloped and degenerates during fetal development. Our 3D morphometric and serial histological analyses have not identified any sign of the allantoic gut throughout mouse embryonic stages. The lack of allantoic gut strongly suggests that the bladder is less likely to originate from the extra-embryonic allantois in mice.

We show that the outgrowth extends into the triangular area located rostral to the cloaca. This previously uncharacterized triangular mesenchymal domain is flanked by the umbilical arteries and marked by *Wnt2* gene expression. The rostral outgrowth expands and becomes the bladder in mice (∼E13.5) and humans (∼CS18). Concomitant with bladder formation, we show that the umbilical arteries undergo dramatic remodeling: the MUR degenerates while the LUR emerges. Remodeling of the umbilical arteries occurs in both human and mouse and takes place at comparable embryonic stages, implying a conserved mechanism underlying the co-development.

Our findings suggest, for the first time, that the cloacal outgrowth epithelial signal(s) regulates co-development of the bladder and umbilical arteries. This is in part mediated by the *Shh-Wnt2* genetic pathway. Similarly, the *Shh-Wnt* genetic pathway also regulates the caudal outgrowth of the cloaca and formation of the genital tubercle. Moreover, constitutive activation of the canonical Wnt signaling pathway partially rescues the genital tubercle outgrowth defect of the *Shh* mutants (Miyagawa et al., 2009). It remains to be determined, however, whether *Shh* regulates *Wnt2* directly and/or indirectly because *Shh* mutants exhibit a severe hypoplastic phenotype. It is worth noting that the phenotype of *Shh* mutants is much more severe than that of *Wnt2* mutants and low levels of *Wnt2* transcripts are detectable in *Shh* mutants, which collectively suggest that *Wnt2* is one of many downstream effectors of *Shh* and *Wnt2* expression is also regulated by other signals besides *Shh* (Fig. 7)*.*

A reciprocal relationship between Shh and Wnt2 signals is known to regulate co-development of the cardiac and pulmonary structures (Steimle et al., 2018), whereby *Shh* expression in the foregut epithelial depends on *Wnt* signals from the cardiopulmonary mesenchymal progenitors. *Shh* expression in the cloacal epithelial is not affected in *Wnt2* mutants. This is likely due to *Wnt2b,* a highly conserved and functionally related *Wnt2* paralog that is also expressed in the bladder mesenchyme (Visel et al., 2004). *Wnt2* and *Wnt2b* function redundantly during lung development (Goss et al., 2009; Peng et al., 2013). We suspect that both *Wnt2* and *Wnt2b* are required to induce *Shh* expression in the cloacal rostral outgrowth. Alternatively, it is worth noting that umbilical artery development precedes cloacal outgrowth during mouse embryogenesis. Intriguingly, bladder formation is significantly affected by complications in twin pregnancies with shared placenta (Wohlmuth and Gardiner, 2022). Future studies are needed to examine whether putative signal(s) from the umbilical arteries induce rostral outgrowth of the cloaca.

The proposed moonlighting role of the embryonic bladder predicts that lack of a bladder is incompatible to life. The onset of bladder development and umbilical artery remodeling takes place during the second month of gestation in humans. It is worth noting that nearly 80% of miscarriages in human pregnancies occur in the first trimester with unknown etiology (Wilcox et al., 1988; Ammon Avalos et al., 2012; Mukherjee et al., 2013). An impaired co-development program might be an overlooked reason for those miscarriages. Future studies are needed to determine the extent to which the molecular network underlying normal bladder development may contribute to development of the umbilical arteries and, ultimately, survival of the mammalian conceptus and successful pregnancies.

## MATERIALS AND METHODS

### Mice

All animal studies were reviewed and approved by the Institutional Animal Care and Use Committee at Cedars-Sinai Medical Center. Wild-type C57BL/6 mice were obtained from Charles River Laboratories. *Shh^+/−^* [B6.Cg-*Shh^tm1(EGFP/cre)Cjt/J^* (005622) and *R26R^lacZ^* B6.129S4-*Gt(ROSA)26^Sortm1Sor/J^* (003474)] were obtained from The Jackson Laboratory. The *Wnt2* mouse line, as previously described (Goss et al., 2009), was generously provided by Dr Edward Morrisey from the University of Pennsylvania, PA, USA.

### HREM and 3D reconstruction

HREM was performed as previously described (Huang et al., 2016). Briefly, staged mouse embryos (wild type, *Shh^−/−^* and *Wnt2^−/−^*) were fixed in 10% buffer formalin (>1 day) and embedded in JB-4 embedding Kit (Polyscience) containing Eosin (Fisher Scientific) and Acridine Orange (Sigma-Aldrich). High-resolution serial block-face images were collected using a stereo zoom microscope (Zeiss, Axio Zoom.V16) using a Hamamatsu digital camera (Photonics K.K., C11440-10C) from both green and red fluorescent channels. All images were inverted using Photoshop (Adobe, v13.0) and further converted to a volume data using 3D visualization software (Amira v5.4.5) to directly visualize the size and morphometric features of each specimen. Amira files of digitized human embryos from CS8 to CS21, as reported previously (de Bakker et al., 2016), were downloaded directly from http://3datlasofhumanembryology.com. Structures, including the cloaca, bladder and umbilical arteries, were manually annotated by experienced investigators with relevant anatomical expertise. Structures-of-interest were identified based on established anatomical landmarks and the clear light–dark contrast characteristic of HREM imaging and were manually delineated using the ‘lasso’ and ‘brush’ tools in the Segmentation Editor (LabelField module). The annotation was refined by cross-referencing virtual sections at all three *xyz* axes to correct potential errors as indicated by inconsistency in 3D reconstruction. The resulting structural segmentations were used in quantitative measurements including distance, surface area, and volume. Animations were converted into movie files using the MovieMaker module.

### Histology, immunostaining, and whole-mount RNA *in situ* hybridization

The same procedures that we described previously were used (Guo et al., 2014, 2017). Briefly, mouse embryos were micro-dissected in cold PBS and fixed in 10% buffer formalin for the subsequent analyses. Hematoxylin and Eosin staining was performed on serial histological sections of E8.5 embryos. Midline sagittal sections of E15.5 wild-type and *Wnt2^−/−^* embryos were stained using pHH3 (Upstate, 06-570) and smooth muscle actin (SMA; Sigma-Aldrich, A2547) antibodies. Whole-mount RNA *in situ* hybridization of staged embryos was performed using *Wnt2-* and *Shh-*specific riboprobes as previously described (Guo et al., 2017; Goss et al., 2009). Expression patterns of the representative signature genes were manually curated using the publicly available GenePaint database (https://gp3.mpg.de) (Visel et al., 2004).

## Supplementary Material



10.1242/develop.205398_sup1 Supplementary information
