# Supplementary material for: The cloacal outgrowth orchestrates co-development of the bladder and umbilical arteries
Source: Development. 2026 Aug 5;153(15):dev205398. doi: 10.1242/dev.205398 (PMC13480954; doi:10.1242/dev.205398)
Supplement: Supplementary file 1 [file develop-153-205398-s1.pdf]

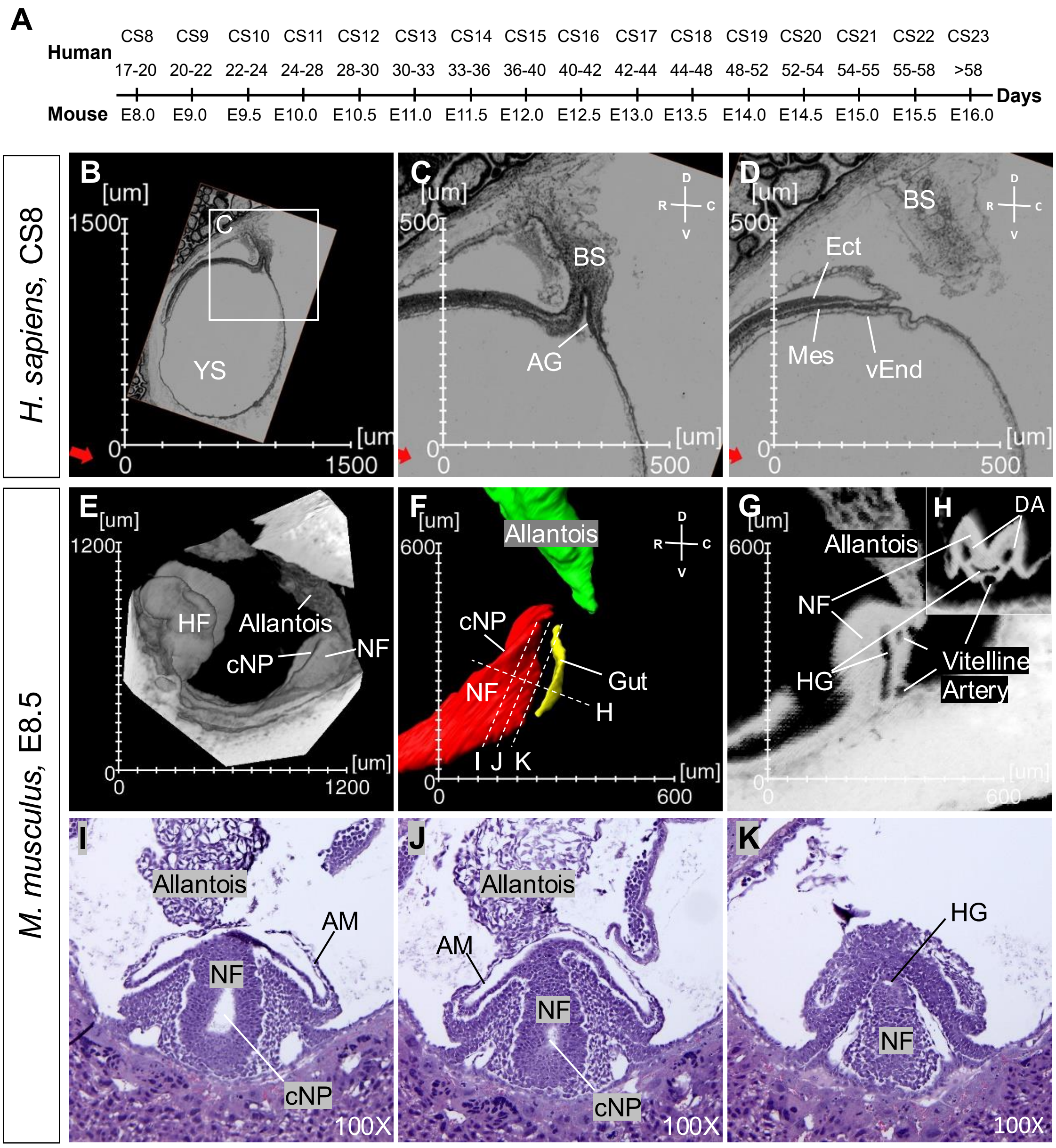

Fig. S1. Related to Figure 1. Unlike humans, mouse embryos do not have the allantoic gut. A, Comparative developmental stages of human and mouse embryos. CS, Carnegie stage; E, embryonic day. **B-D**, Digital sagittal sections of CS8 human embryo through the allantois (B and C) and at the lateral side (D). **E-G**, Digital sagittal views of wholemount (E), 3D segmentation (F), and midline section (G) of E8.5 mouse embryo. **H**, Digital cross section of E8.5 embryos from the plane shown in F. **I-K**, Serial histologically stained sections of E8.5 mouse embryos. Locations and orientations of the sections are shown in F. AM, amnion; BS, body stalk; cNP, caudal neural pore; Ect, ectoderm; HF, headfold; Mes, mesoderm; vEnd, visceral endoderm; NF, neural fold; YS, yolk sac. See Figure 1 for additional abbreviations.

Development • Supplementary information

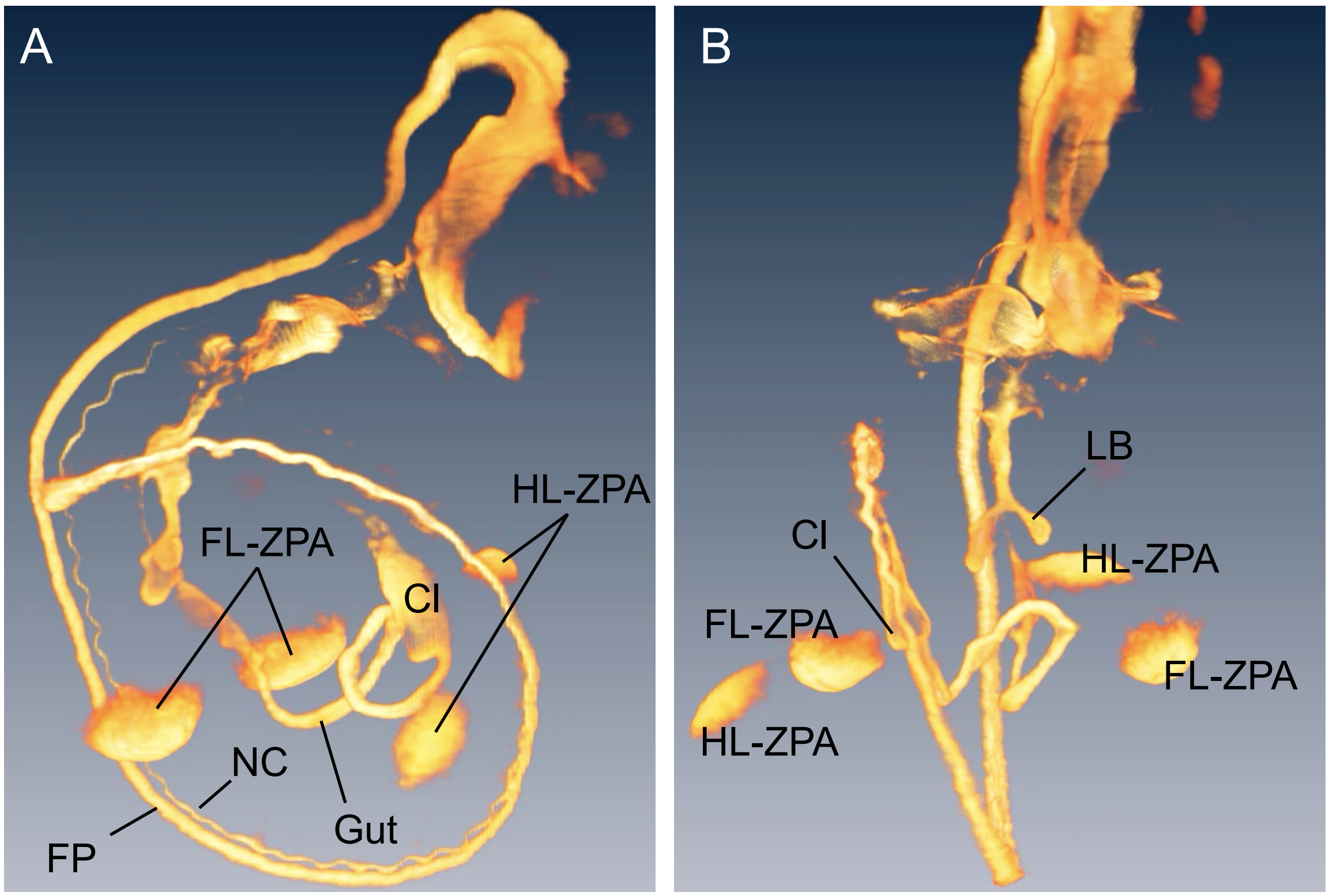

Fig. S2. Related to Figure 4 and Movie 5. Distribution of the genetic lineages of *Shh*-expressing cells. Double heterozygous *Shh*$^{+/-}$;*R26R*^{lacZ} embryos (E10.5) were stained with X-gal to detect the *Shh*-expressing genetic lineages. The *Shh*$^{+/-}$ mutant allele expresses Cre recombinase under the control of endogenous *Shh* promoter and indelibly labels all *Shh*-expressing cells and their progenies. Stained cells were visualized directly using HREM. Sagittal (A) and frontal (B) 2D images were taken (related to Movie 5). Cl, cloaca; FL, forelimb bud; FP, floor plate of the spinal cord; HL, hindlimb bud; LB, lung bud; ZPA, zone of polarizing activity of the limb buds.

Development • Supplementary information

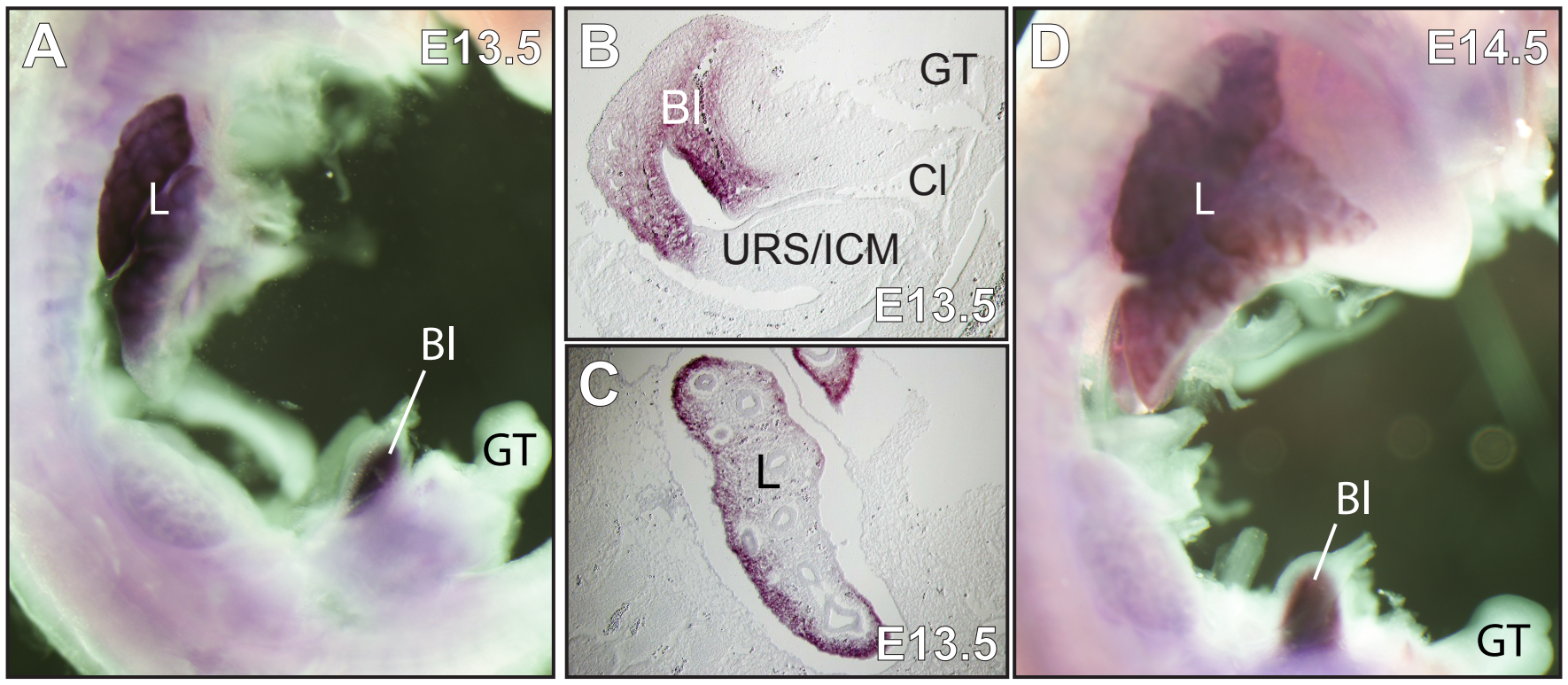

Fig. S3. Related to Figure 5. *Wnt2* is specifically expressed in mesenchymal progenitors of the developing bladder and lung. Wholemount RNA *in situ* hybridization using a *Wnt2*-specific probe (dark purple staining). **A-C**, A wholemount (A) and cryostat section (B and C) views of E13.5 mouse embryo after *Wnt2 in situ* hybridization. **D**, A wholemount view of E14.5 embryos after *Wnt2 in situ* hybridization.

Table S1. List of HREM digitized human embryos, related to Figure 1

CS	Specimen#	Origin	Year	CRL (mm)	Day	Sex	Plane	Sections#	Pixel size (µm)	Z-res (µm)
8	8671	CC	1949	0.61			T	226	0.86	2.70
9	H712	BC	1957	1.57			T	437	1.81	3.59
10	6330	CC	1931	1.95	28		T	168	0.54	11.63
11	6784	CC	1933	2.46			T	283	0.86	8.70
12	8505A	CC	1947	2.86			T	277	1.08	10.32
13	836	CC	1914	4.09	32	F	T	247	5.89	16.55
14	8314	CC	1945	5.16	22		T	639	2.23	8.07
15	3512	CC	1921	6.55			T	651	2.23	10.06
16	6517	CC	1931	10.46	39		T	547	5.40	19.13
17	6521	CC	1933	10.60			T	1059	4.48	10.01
18	6524	CC	1933	9.73			T	956	4.48	10.18
20	462	CC	1910	15.93		M	T	376	4.48	42.36
21	7254	CC	1936	17.36		M	T	288	4.48	60.12

CS: Carnegie stage; Origin of the specimen: CC: Carnegie Collection, BC: Boyd Collection; Year: Year of acquisition; CRL: Crown-rump-length in mm; Day: days post ovulation; Plane of sectioning: O = Oblique, T= Transversal; Z-res: Calculated Z-resolution in µm.

de Bakker BS, de Jong KH, Hagoort J, de Bree K, Besselink CT, de Kanter FE, Veldhuis T, Bais B, Schildmeijer R, Ruijter JM, Oostra RJ, Christoffels VM, Moorman AF. An interactive three-dimensional digital atlas and quantitative database of human development. Science. 2016 Nov 25; 354 (6315): aag0053.

Table S2. List of HREM digitized mouse embryos, related to Figures 1-3

Specimen#	Embryonic days	Somite#	Sections#	Pixel size(µm)	Z-res (µm)
5-6	8.5(2X with YS)		3810	3.63	2.00
122	9.5a	27	1567	1.80	1.50
123	9.5b	27	1492	1.50	1.50
124	9.5c	27	1446	1.60	1.50
1908	10.0a	31	1740	1.50	1.50
145	10.0b	30	1742	1.70	1.50
147	10.0c	32	1811	1.60	1.50
78	10.5a	36	1995	2.10	2.00
42	10.5b	35	1458	2.10	2.10
37	10.5c	37	1650	2.00	2.00
57	11.0a	41	2179	2.40	2.00
58	11.0b	43	2374	2.50	2.00
56	11.5a	46	2477	3.00	2.00
83	11.5b	45	2349	2.30	2.00
66	11.5c	46	2682	2.80	2.00
125	12.0a	48	2602	2.80	2.00
86	12.0b	48	2578	2.60	2.00
126	12.0c	48	2776	2.40	2.00
34	12.5a	>50	2533	3.20	2.50
89	12.5b	>50	2552	2.90	2.50
128	12.5c	>50	2797	2.90	2.50
141	13.0a	>55	3247	3.20	2.50
142	13.0b	>55	3237	3.50	2.50
157	13.0c	>55	3511	3.20	2.50
72	13.5a	>55	3006	3.80	2.50
73	13.5b	>55	2997	3.60	2.50
77	13.5c	>55	3359	3.80	2.50

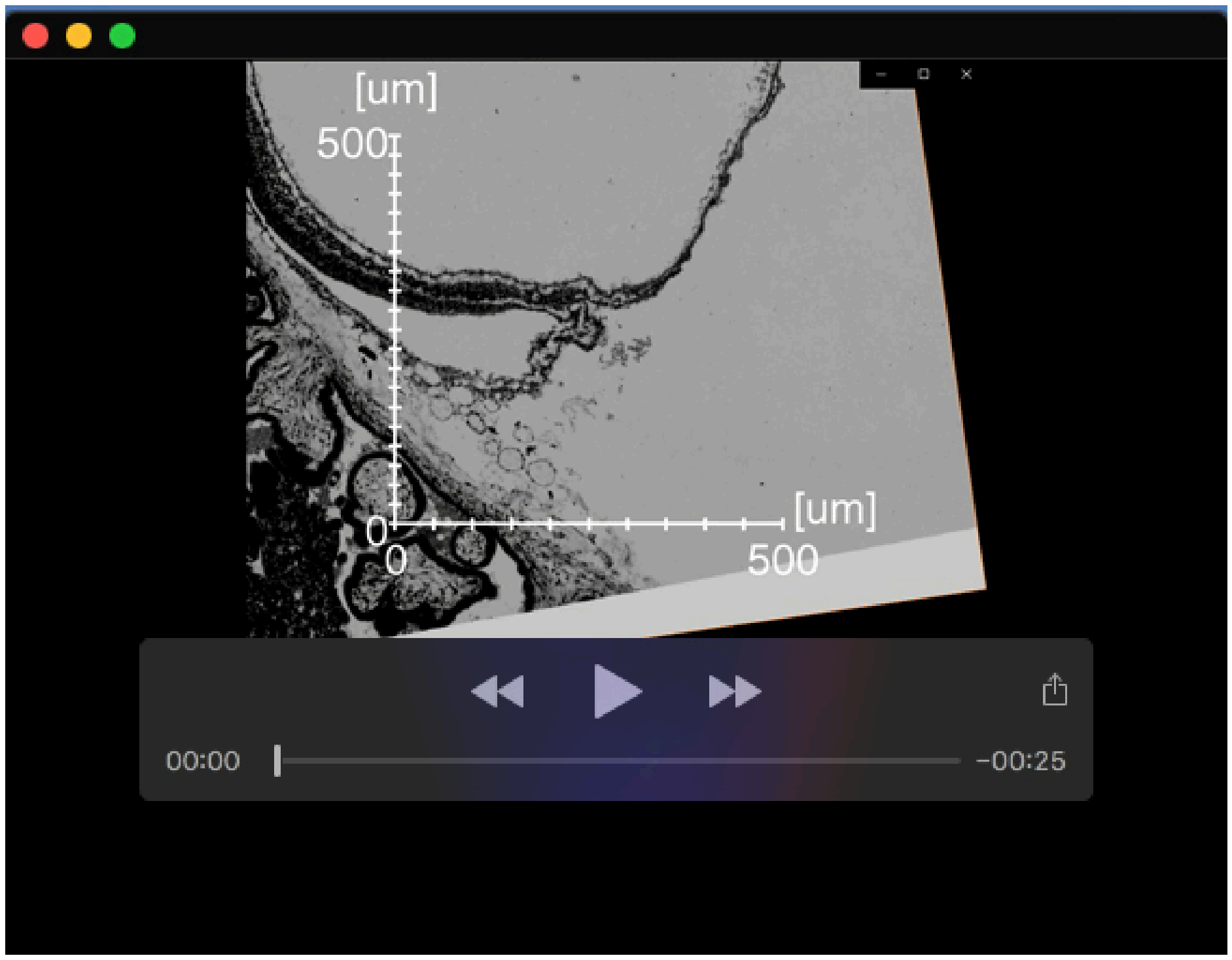

Movie 1. Video related to Figure S1. **2D animation of virtual sagittal sections through the entire allantois of CS8 human embryos**.

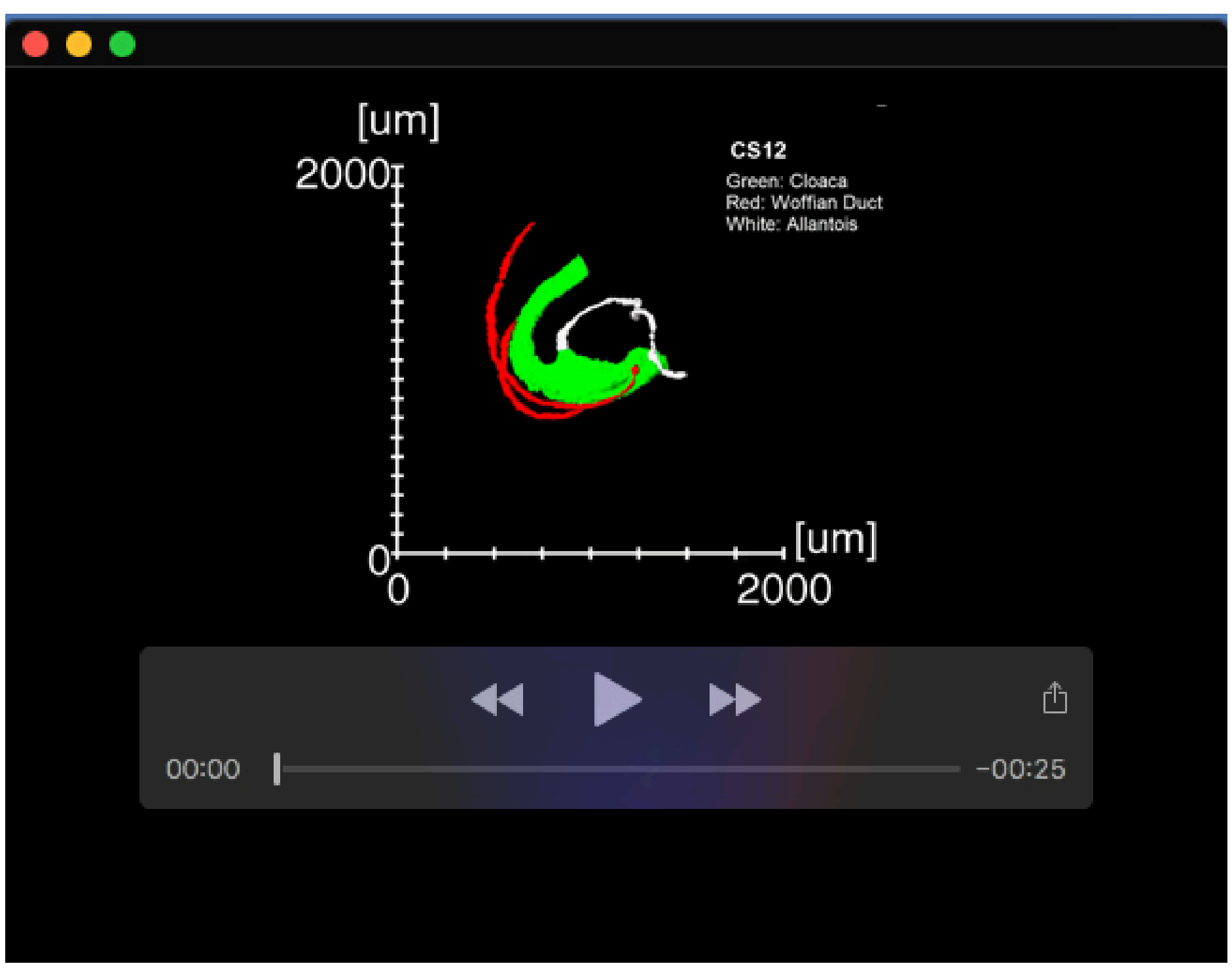

Movie 2. Video related to Figure 1. **3D visualization of the developing urinary tract and the associated arterial network of human embryos** at CS12, 14, 18, and 21.

Development • Supplementary information

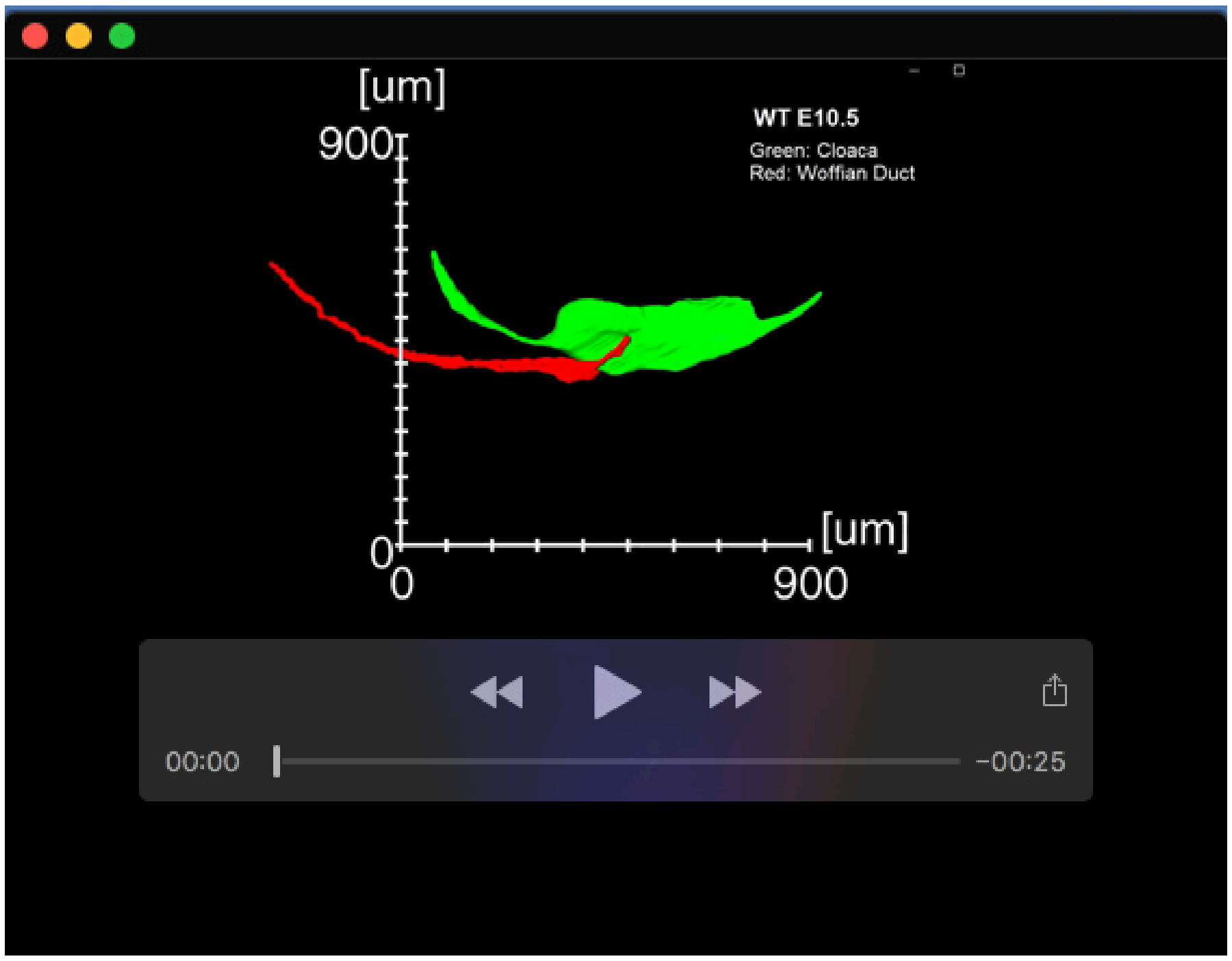

Movie 3. Video related to Figures 1-3. 3D visualization of the developing mouse cloaca and the associated arterial network from E10.5, 11.5, 13.5, to E15.5.

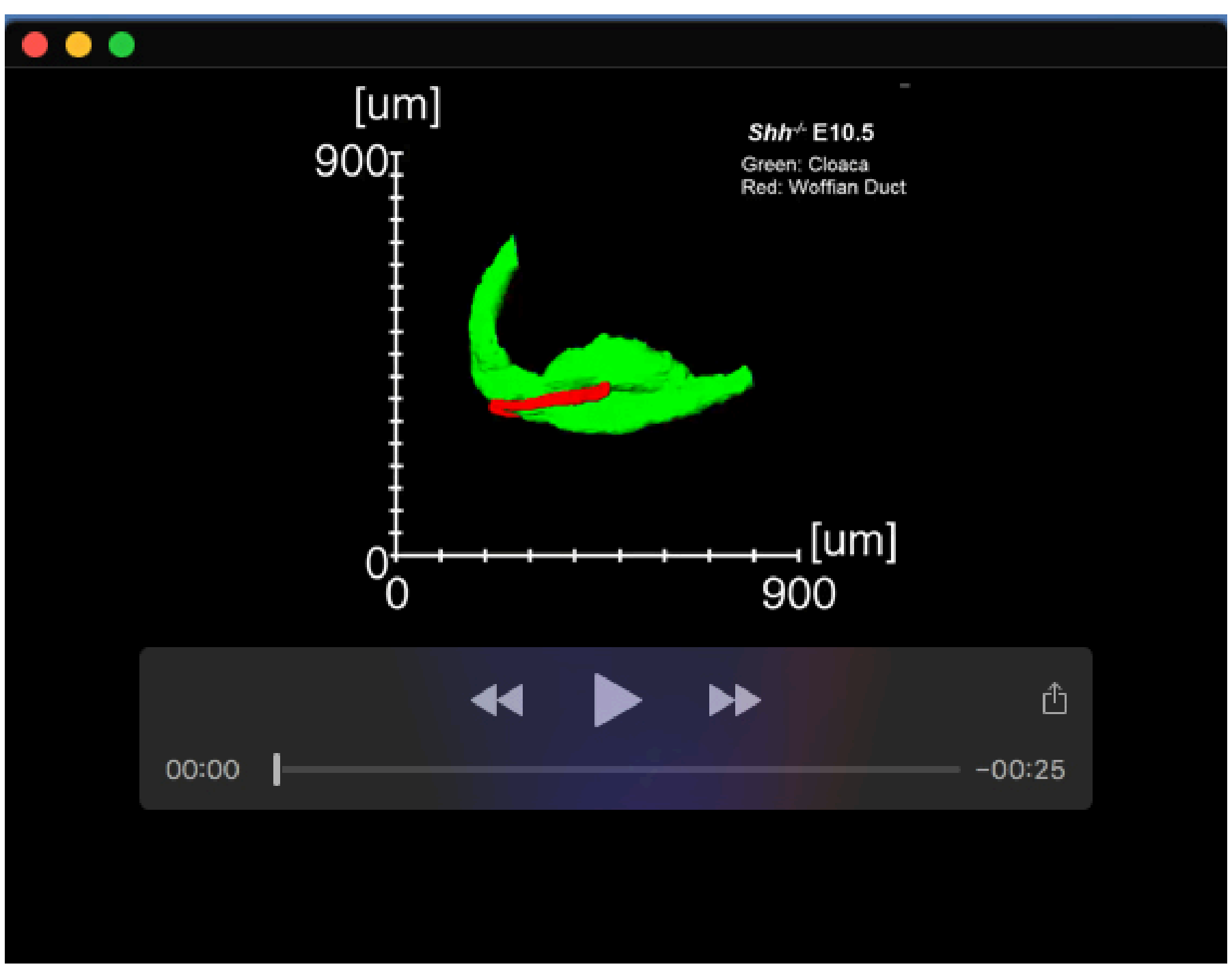

Movie 4. Video related to Figure 4. 3D visualization of the developmental defects of the bladder and umbilical arteries of *Shh*$^{-/-}$ mutant mouse embryos at E10.5, 11.5, 12.5, and E13.5. *, indicating absence of the bladder.

Development • Supplementary information

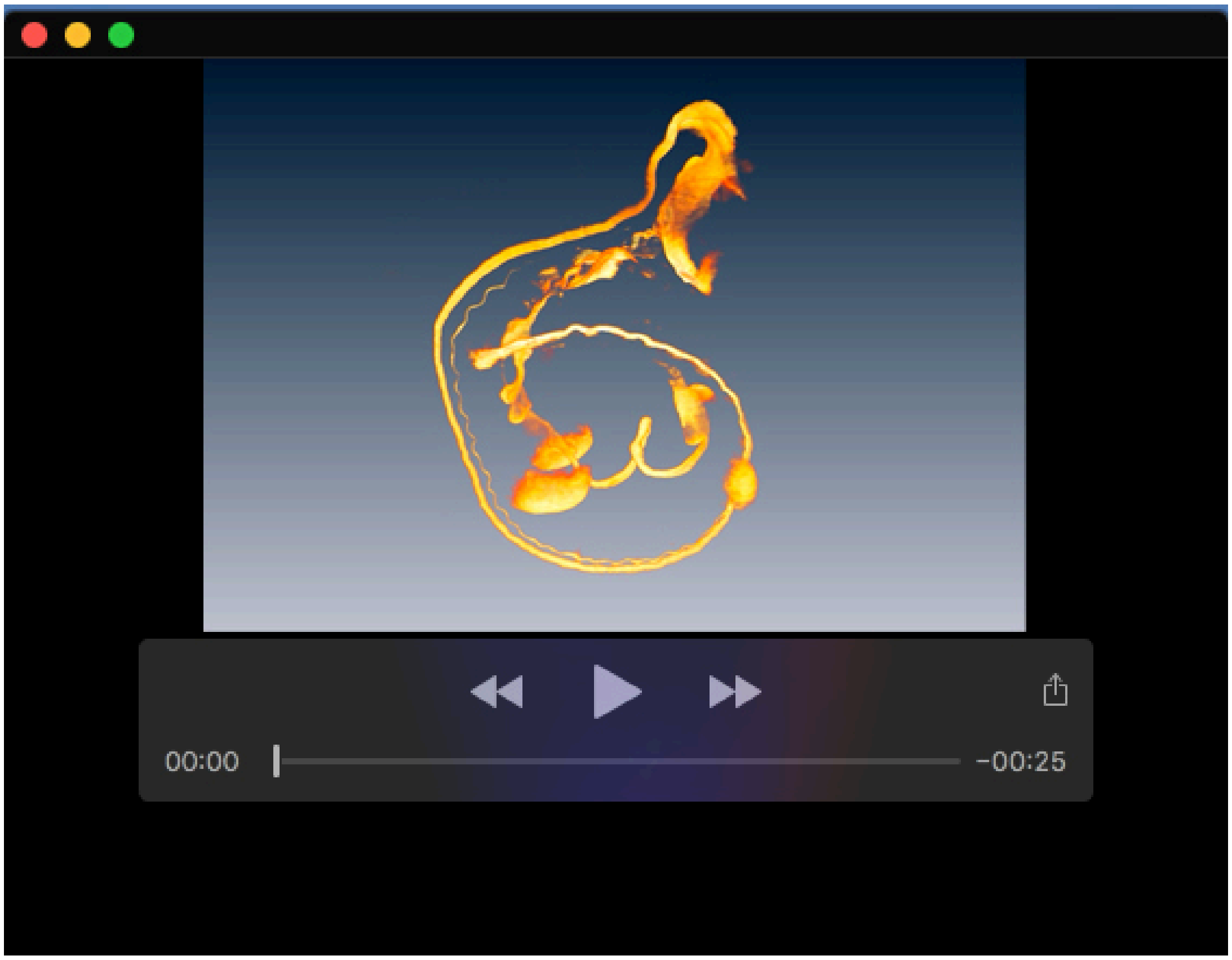

Movie 5. Video related to Figure 4. 3D visualization of the *Shh*-positive (*Shh*$^{+}$) lineages at E11.5 (N=2). Double heterozygous *Shh*$^{+/-}$*;R26R*^{lacZ} embryos were stained with X-gal to detect the Shh^{+} lineages. Stained cells were visualized directly using HREM to create the 3D views.

Development • Supplementary information
