# Supplementary material for: The cloacal outgrowth orchestrates co-development of the bladder and umbilical arteries
Source: Development. 2026 Aug 5;153(15):dev205398. doi: 10.1242/dev.205398 (PMC13480954; doi:10.1242/dev.205398)
Supplement: Supplementary file 2 [file develop-153-205398_review_history.pdf]

The cloacal outgrowth orchestrates co-development of the bladder and the umbilical arteries

Xing Ye, Liguang Xia, Xianfa Yang, Ruirong Tan, Haochuan Zhang, Liming Lei, Jungang Huang, Yingsheng Zhang, Edward Morrisey, Ping Zhu, Zhongrong Li, Naihe Jing and Xue Li
DOI: 10.1242/dev.205398

Editor: Anna-Katerina Hadjantonakis

Review timeline

Original submission:	24 November 2025
Editorial decision:	5 January 2026
First revision received:	8 April 2026
Editorial decision:	27 April 2026
Second revision received:	17 June 2026
Accepted:	25 June 2026

Original submission

First decision letter

MS ID#: dev.205398

MS TITLE: The cloacal outgrowth orchestrates co-development of the bladder and umbilical arteries

AUTHORS: Xing Ye, Liguang Xia, Xianfa Yang, Ruirong Tan, Haochuan Zhang, Liming Lei, Jungang Huang, Yingsheng Zhang, Edward Morrisey, Ping Zhu, Zhongrong Li, Naihe Jing and Xue Li

Dear Dr Li,

I have now received all the referees' reports on the above manuscript, and have reached a decision. The referees' comments are appended below.

As you will see, the referees express considerable interest in your work, but have some significant criticisms and recommend a substantial revision of your manuscript before we can consider publication. If you are able to revise the manuscript along the lines suggested, which may involve further experiments, I will be happy to receive a revised version of the manuscript. Your revised paper will be re-reviewed by one or more of the original referees, and acceptance of your manuscript will depend on your addressing satisfactorily the reviewers' major concerns. Please also note that Development will normally permit only one round of major revision. If it would be helpful, you are welcome to contact us to discuss your revision in greater detail. Please send us a point-by-point response indicating your plans for addressing the referees' comments, and we will look over this and provide further guidance.

Please attend to all of the reviewers' comments and ensure that you upload both a 'clean' version of your Word file, along with a highlighted version clearly showing where you have made changes in the revised manuscript. Please avoid using 'Tracked changes' in Word files as these are lost in PDF conversion. I should be grateful if you would also provide a point-by-point response detailing how you have dealt with the points raised by the reviewers in the 'Response to Reviewers' box. If you do not agree with any of their criticisms or suggestions please explain clearly why this is so.

Reviewer 1

© 2026. Published by The Company of Biologists under the terms of the Creative Commons Attribution License (https://creativecommons.org/licenses/by/4.0/).

SUMMARY OF THE ADVANCE MADE IN THIS PAPER AND ITS POTENTIAL SIGNIFICANCE TO THE FIELD

In this study, the authors investigated the role that the cloaca plays in the co-development of the bladder and umbilical arteries. Using HREM, the authors first described that the bladder develops from the cloaca and the vascular remodeling of umbilical arteries during the rostral outgrowth of the cloaca. The authors then reported severe developmental defects in Shh-/- mice and Wnt2-/- mice, suggesting an important Shh-Wnt2 axis-mediated mechanism in the development of the bladder and umbilical arteries. The findings of this study can elucidate key cross-talk between these two organs and deepen understanding of clinical birth defects or even miscarriages.

SUGGESTIONS TO AUTHORS

Major Suggestions:

1. In general for HREM studies, it would be helpful to provide more detail on how structures are manually annotated and provide additional data (e.g., H&E and/or lineage-specific immunofluorescence/IHC) to support the accuracy of the manual annotation. More specific instances are mentioned in detailed comments below.
2. The article could be improved with more in-text reporting of quantification and statistical analysis (N, SD). More specific instances are mentioned in detailed comments below.
3. For Figures 1 and S1, please consider adding the number of murine embryos examined and indicating whether the HREM findings are consistent across all samples.
4. For Figure 2, please consider providing the N and SD for the statistical analysis in panels G and H.
5. The authors suggest that the entire urinary bladder originates from the cloaca without any contribution from the allantois based on serial HREM imaging. It would be helpful to further support this conclusion with lineage tracing studies—either showing that the entire bladder can be lineage-traced back to the cloaca or that the allantois lineage does not contribute to the bladder—to rule out the possibility of cell migration during development.
6. For Figure 3, please consider adding a caudal view for the human samples.
7. For the section titled "The Rostral Outgrowth Links Development of the Bladder and Umbilical Arteries" (line 202), the reviewer does not feel that the results presented in lines 203-223 sufficiently support this conclusion. In this section, the authors described observations of umbilical artery remodeling during the rostral outgrowth of the cloaca. While the conclusion may be true, this section does not contain sufficient evidence to establish a link between umbilical artery development and bladder development.
8. For lines 230-235, please consider providing additional data such as immunostaining and the number of samples examined to support the conclusion that bladder development is absent. It would be helpful to subsequently interpret the result in the context of findings by Perriton et al. and Haraguchi et al., who found that in Shh-/- mice, the bladder develops although severely hypoplastic.
9. For lines 245-246, it would be helpful to provide immunostaining data to show that Shh-lineage and the umbilical artery are mutually exclusive.
10. For Figure 4 and its related results section, please consider adding the number of samples examined and indicating whether findings were consistent across all samples.
11. For line 252, please consider adding quantification regarding umbilical artery size in Shh-/- mice compared to controls.
12. Regarding the vascular anomaly in Wnt2-/- mice (Figures 6A-D, lines 294-299), please consider adding the number of samples examined and stating whether findings were consistent across all samples.
13. Regarding Figures 6E-H, is there a reason the authors chose E12.5 for comparison to E15.5 in Figures 6A-D? Could the low Wnt2 expression represent a developmental delay rather than a defect? The reviewer also wonders whether the weaker Wnt2 signal is due to lower expression levels in each cell or due to hypoblastic mesenchyme in Shh-/- animals.
14. Regarding Figures 6E-H, have the authors considered performing the same staining in Wnt2-/- animals?
15. Regarding the proposed model in Figure 7: if Shh signaling works through Wnt2, why do Shh-/- animals exhibit seemingly more severe developmental defects for both bladder and umbilical artery development compared to Wnt2-/- animals? For example, Wnt2-/- animal has hypoplastic bladder vs. bladder agenesis in Shh-/-; Wnt2-/- animal develops LUR but Shh-/- does not.

© 2026. Published by The Company of Biologists under the terms of the Creative Commons Attribution License (https://creativecommons.org/licenses/by/4.0/).

16. The idea that Shh and Wnt signaling affect umbilical artery development is very interesting. Have the authors considered studying whether the umbilical artery or MUR/LUR express Shh or Wnt2 receptors and response genes, as the arrows in the schematic might suggest in Figure 7? If so, what is the spatial and temporal expression pattern during development? This could help elucidate whether the Shh-Wnt2 signaling occurs directly from the cloaca or through some other intermediate tissue or organs.

Minor Suggestions:
1. For HREM images in the figures, please consider color-coding the labels to match the color of the corresponding organ to help the reader.
2. For all figures, please consider including all abbreviations in the figure legend, not only those new to each figure. For example, mention what Cl and TG stand for in Figure 2 even though they appeared in Figure 1.
3. Please consider adding explanations of what the arrows in Figure 3A and 3B indicate in within the figure legend.
4. For Figure S5, please consider annotating key anatomical structures to orient the readers.
5. For lines 266-267, please consider adding data and an explanation regarding the significance of Wnt2 in your analysis; for example, clarify that Wnt2 was the top candidate in what particular analysis.
6. It would be helpful for the authors to discuss why, in Shh-/- and Wnt2-/- animals, one umbilical artery still develops while the other does not. The reviewer is also curious if it is always one particular side (e.g. right side) that develops in each model.
7. It would be helpful for the authors to discuss whether, in addition to cloaca→umbilical artery signaling, there may be umbilical artery→cloaca signaling involved in bladder development.
8. It would be helpful for the authors to mention if developmental defects in bladder has been associated with vascular anomaly in human, if such data is available.

<u>Reviewer 2</u>

Comments for the author

Bladder development remains poorly understood.
The dev.205398 describes co-developmental processes of the bladder and umbilical arteries It also shows such co-development is orchestrated by the outgrowth epithelial signal Shh and mesenchymal signal Wnt2. The MS also contains human embryonic data offering insights for the unexplored topics.

The work is compactly summarized and I basically support its publication.

I have several points to potentially improve the MS.

Wnt2 phenotypes;
The Wnt2 deficient bladders are smaller indeed but the phenotypes are not robust. Any possibility of other Wnts involvement ?

Shh-Wnt2 signals;
Shh coordinates development of both the bladder and umbilical arteries. Wnt2 signaling is restricted to the rostral outgrowth mesenchyme with its expression down-regulated in Shh-null mutants.
Is the signal reciprocal in a way of Wnt2 (mesen) to epithelial Shh? In the middle of Fig.7, it in fact shows Shh to Wnt by arrows. But the left Fig shows Wnt2(green) to Shh (red)?

There is such case of reciprocal signal for the co development of the lungs and heart with Wnt2 and Shh. This can be mentioned. https://doi.org/10.1073/pnas.1811624115

Detailed descriptions in Fig 1-3;
Detailed data including human embryos are effective to elucidate the coordinated organs development.

© 2026. Published by The Company of Biologists under the terms of the Creative Commons Attribution License (https://creativecommons.org/licenses/by/4.0/).

As for its implications for human birth defects, it has been known that Bladder formation is significantly affected by complications in twin pregnancies with shared placentas and other structural urinary defects can occur compared with singleton. Comments on such based on the current discussions? Such will appeal to wide range of Development readers.

Mice are known for pregnancy with multiple litter size. In their data, Unlike human embryos, an expected endodermal protrusion was never observed in mouse embryos. However, remodeling of the umbilical arteries is detected in both species.
Speculation or Species related comments could be also described?

Related Shh-Wnt signals around cloaca;
About the Wnt-Shh interaction with cloaca and genital tubercle, there is a previous study with Shh (loss) and Wnt (gain of function) mutant analysis. ß-catenin GOF mutation rescues defective GT outgrowth from the cloaca.
https://doi.org/10.1242/dev.039438

In sum,the work is effective in elucidating complex coordinated organs development with appropriate revision.

Reviewer 3

SUMMARY OF THE ADVANCE MADE IN THIS PAPER AND ITS POTENTIAL SIGNIFICANCE TO THE FIELD

This is a very interesting study focused on the role of the cloaca, the primordium of the bladder, urethra and gut, as an organizing center that promotes normal patterning of umbilical arteries. The authors use mutants lacking Wnt2 and Shh in their studies to elucidate the functions that underlie interactions between the cloaca and Umbilical arteries.

Comments/Suggestions

It would be good to include description of the structure of the Umbilical arteries and their derivation to aid readers in understanding the process.
The visual and textual descriptions of UA formation are very difficult to follow as written. (ie muscle endothelial cells). Related to this, are all of the components derived from the same embryonic primordia? Is there a possible connection between extraembryonic mesoderm and cloacal mesoderm? There have been some studies looking at the derivation of the mesodermal component of the cloaca-it would be good to include some of this data in discussions or in the introduction to evaluate a possible link between cloacal mesoderm and extra-embryonic mesoderm as above.

The embryonic/adult umbilical arteries are well studied, and it is generally accepted that the umbilical artery is derived from extraembryonic mesoderm while the epithelial component of the cloaca is derived from endoderm. As the allantois is not prominent in mice as it is in humans, the issue of cloaca derivation from the allantois is not really present in the mouse field, but is discussed mostly in older studies looking at human tissue/fetal malformations. However, one could argue that things are different in humans vs mouse, and that the embryonic origins of the allantois and cloacal tissue are more closely linked in humans vs mice. This issue is not addressed experimentally here, but could be for example by trajectory analysis of human developing embryos using sc RNA-seq for example, or spatial RNA-seq analysis of Paraffin embedded material from human embryos.

Shh drives an important signaling center in the midline. Its absence leads to many midline-related and laterality abnormalities in the brain, eyes, head/neck skeleton. Shh mutants also exhibit defective cloacal septation and defective bladder differentiation, as well as imperforate anus. One could argue that it is the absence of Shh signaling center is the the major issue, as opposed to 'the cloaca". One could imagine that abnormal tissue size or position for example failure in cloacal septation is going to affect 3-D patterning of structures including the UAs which are closely associated with the bladder, resulting in defects such as single UA. To prove that the cloaca is the source of signals one would have to either identify the cloacal-derived signal (is it Shh or Wnt2?) or

© 2026. Published by The Company of Biologists under the terms of the Creative Commons Attribution License (https://creativecommons.org/licenses/by/4.0/).

evaluate whether UA form when there is cloacal agenesis. Along these lines Is the single umbilical artery phenotype found in individuals without anomalies in cloacal derived structures? This can't really be done from observation of sections or 3D analysis.

How does Bmp7 fit in here? Previous studies from other groups suggest that Shh cooperates with Bmp signaling to orchestrate events during cloacal differentiation. The authors should comment on this.

First revision

Author response to reviewers' comments

> *I want to sincerely thank the reviewers for their careful reviews of this manuscript. They each asked interesting and insightful questions and pointed out limitations. I enjoyed thinking about the revisions and believe I have addressed each of the comments. I think it has made a world of difference, and I am grateful for the feedback and suggestions.*

One-to-one rebuttal:

Reviewer 1: In this study, the authors investigated the role that the cloaca plays in the co-development of the bladder and umbilical arteries. Using HREM, the authors first described that the bladder develops from the cloaca and the vascular remodeling of umbilical arteries during the rostral outgrowth of the cloaca. The authors then reported severe developmental defects in Shh-/- mice and Wnt2-/-mice, suggesting an important Shh-Wnt2 axis-mediated mechanism in the development of the bladder and umbilical arteries. The findings of this study can elucidate key cross-talk between these two organs and deepen understanding of clinical birth defects or even miscarriages.

> *Response:* We thank the Reviewer for the in-depth review, insightful comments, and constructively suggestions, which have clearly improved the revised manuscript.

Major Suggestions:

1. In general for HREM studies, it would be helpful to provide more detail on how structures are manually annotated and provide additional data (e.g., H&E and/or lineage-specific immunofluorescence/IHC) to support the accuracy of the manual annotation. More specific instances are mentioned in detailed comments below.

> *Response:* We thank the reviewer for this helpful comment. We have included the following description in the revision (line 420-427). Representative 2D images are included in the Supplementary Material to support the accuracy and consistency of the annotation strategy (Figure S1B-D and new Figure S2). To ascertain the annotation, selected structures were confirmed independently using high-resolution serial histological sections (Figure S1_K).
>
> "Manual annotation of HREM images was conducted by experienced investigators with relevant anatomical expertise. Structures were identified based on established anatomical landmarks and the clear light–dark contrast characteristic of HREM imaging and were manually delineated using the lasso and brush tools in the Segmentation Editor (LabelField module). 3D reconstruction was done initially based on annotations performed on the original high-resolution images. The annotation was refined by cross-referencing virtual sections at all three XYZ axes to correct potential errors as indicated by inconsistency in 3D reconstruction."

2. The article could be improved with more in-text reporting of quantification and statistical analysis (N, SD). More specific instances are mentioned in detailed comments below.

> *Response:* We appreciated the suggestion. Sample sizes were included in supplemental Table S1, which have also been included in the revised Figure legends.

© 2026. Published by The Company of Biologists under the terms of the Creative Commons Attribution License (https://creativecommons.org/licenses/by/4.0/).

3. For Figures 1 and S1, please consider adding the number of murine embryos examined and indicating whether the HREM findings are consistent across all samples.

Response: We updated the figure legend to include the data.

4. For Figure 2, please consider providing the N and SD for the statistical analysis in panels G and H.

Response: We updated the figure legend to include the data. Results of Student's *t*-test were also included.

5. The authors suggest that the entire urinary bladder originates from the cloaca without any contribution from the allantois based on serial HREM imaging. It would be helpful to further support this conclusion with lineage tracing studies—either showing that the entire bladder can be lineage-traced back to the cloaca or that the allantois lineage does not contribute to the bladder—to rule out the possibility of cell migration during development.

Response: We thank the reviewer for the insightful comments. As suggested, we used the genetic lineage tracing strategy to indelibly label all the *Shh*-expression progenitors and their progenies. This strategy enables permeant tagging all the *Shh* genetic lineages and the tagged cells can be visualized by a highly specific and sensitive X-gal staining protocol. Moreover, the stained embryos were analyzed using HREM 3D approach, which provides a comprehensive overall view as well as single-cell resolution views. Findings from this analysis strongly suggest that the entire bladder can be lineage-traced back to the cloaca (Figure S6, original S5). However, we are unable to trace allantois lineages because no genetic tools are currently available. Therefore, we could not rule out the possibility that the allantois could de-differentiate into the bladder. We have discussed this limitation in the manuscript (line 337-340).

6. For Figure 3, please consider adding a caudal view for the human samples.

Response: The caudal view was included in the Figure S3 (original S2). Here, a caudal perspective of the human samples is clearly visible in the three-dimensional reconstruction videos, which enable comprehensive visualization of the structures from all orientations.

7. For the section titled "The Rostral Outgrowth Links Development of the Bladder and Umbilical Arteries" (line 202), the reviewer does not feel that the results presented in lines 203-223 sufficiently support this conclusion. In this section, the authors described observations of umbilical artery remodeling during the rostral outgrowth of the cloaca. While the conclusion may be true, this section does not contain sufficient evidence to establish a link between umbilical artery development and bladder development.

Response: We thank the reviewer for this thoughtful comment and have replaced "Links" to "Coincides", intending to soften the claim. Our observations are based on high-resolution serial HREM imaging, which reveal the morphological changes of the cloacal rostral outgrowth, bladder morphogenesis, and umbilical artery remodeling. The spatiotemporal coincidence of these developmental events/structures implies that the rostral outgrowth is tightly associated with development of both the bladder and umbilical arteries. Indeed, this notion is further supported by the mechanistic link that the epithelial SHH signal controlled umbilical artery remodeling (Figure 4). However, future mechanistic studies, such as identifying downstream targets of *Shh* signaling required for umbilical artery remodeling, would further strengthen the model.

8. For lines 230-235, please consider providing additional data such as immunostaining and the number of samples examined to support the conclusion that bladder development is absent. It would be helpful to subsequently interpret the result in the context of findings by Perriton et al. and Haraguchi et al., who found that in Shh-/- mice, the bladder develops although severely hypoplastic.

Response: We thank the reviewer for the insights. We have provided the number of samples examine in the figure legend to support the conclusion. We also addressed

© 2026. Published by The Company of Biologists under the terms of the Creative Commons Attribution License (https://creativecommons.org/licenses/by/4.0/).

the comment of immunostaining below in #9. Regarding the interpretation, we have *revised the paragraph and include additional citations as followings:*

"In addition, the rostral and caudal outgrowth of the UGS was arrested at E12.5, resulting in agenesis of the urinary bladder and genital tubercle as reported previously {Haraguchi, 2001 #626;Perriton, 2002 #643;Haraguchi, 2007 #624}. Stage-specific deletions of *Shh* further indicate an early phase requirement of *Shh* signaling cloacal septation, formation of the bladder and genital tubercle, and a later phase requirement of external genital development {Seifert, 2009 #989;Miyagawa, 2009 #994;Lin, 2009 #3862}. Together, these findings show that epithelial *Shh* signal is critically required for formation of the bladder and genital tubercle." (line 241-247)

9. For lines 245-246, it would be helpful to provide immunostaining data to show that Shh-lineage and the umbilical artery are mutually exclusive.

Response: We used the genetic lineage tracing strategy to indelibly label all the *Shh*-expression progenitors and their progenies. This strategy enables permeant tagging all the *Shh* genetic lineages and the tagged cells can be visualized by a highly specific and sensitive X-gal staining protocol. Moreover, the stained embryos were analyzed using HREM 3D approach, which provides a comprehensive overall view as well as single-cell resolution views. Overall, we believe that these strategies serve as excellent alternatives to immunostaining. We therefore did not perform additional immunostaining.

10. For Figure 4 and its related results section, please consider adding the number of samples examined and indicating whether findings were consistent across all samples.
Number.

Response: We updated the figure legend to include the data.

11. For line 252, please consider adding quantification regarding umbilical artery size in Shh-/- mice compared to controls.

Response: We appreciate the suggestion. We did not measure umbilical artery size separately because it is indicated in the Figure with the X and Y axes scaled. Moreover, the mutant umbilical arteries, the MUR, persists at E11.5. This contrasts sharply with wild type MUR, which degenerates completely. Since wild type MUR disappeared completely, there is nothing left to measure.

12. Regarding the vascular anomaly in Wnt2-/- mice (Figures 6A-D, lines 294-299), please consider adding the number of samples examined and stating whether findings were consistent across all samples.

Response: We updated the figure legend and text to include the data.
We also stated that the findings are consistent across all samples.

13. Regarding Figures 6E-H, is there a reason the authors chose E12.5 for comparison to E15.5 in Figures 6A-D? Could the low Wnt2 expression represent a developmental delay rather than a defect? The reviewer also wonders whether the weaker Wnt2 signal is due to lower expression levels in each cell or due to hypoblastic mesenchyme in Shh-/- animals.

Response: We appreciate the thoughtful comments. To determine whether *Wnt2* regulates growth of the bladder, we choose E15.5 because this is an ideal stage to measure bladder size for the following reasons: 1) the bladder is anatomically defined at this stage. This is critical to accurately separate components the bladder, including the lumen, lamina propria, and the smooth muscle layer. 2) E15.5 embryos do not produce urine, which is a major confound variable of size measurement because urine distends the bladder. To avoid this confounding variable, we therefore choose E15.5.

Comparing to *Wnt2* mutants, *Shh* mutants display much more severe phenotype with bladder development arrested at E12.5. For molecular analysis, we therefore chose E12.5. We show that *Wnt2* expression is dramatically decreased in *Shh* mutants at this stage. While this finding suggest that *Shh* may regulate expression of *Wnt2*. However, we could rule out the possibility that down-regulation of *Wnt2* is secondary to the hypoplastic phenotype. We have noted this in the revised

© 2026. Published by The Company of Biologists under the terms of the Creative Commons Attribution License (https://creativecommons.org/licenses/by/4.0/).

manuscript (line 355-365).

14. Regarding Figures 6E-H, have the authors considered performing the same staining in Wnt2-/- animals?

Response: We have but did not observe any significant change of *Shh* expression in *Wnt2* mutants. Because *Wnt2* and *Wnt2b* have redundant functions, it is possible that deletion of both genes is required to observe any significant alterations of *Shh*. We have discussed this point in the revised manuscript (line 366-378).

15. Regarding the proposed model in Figure 7: if Shh signaling works through Wnt2, why do Shh-/- animals exhibit seemingly more severe developmental defects for both bladder and umbilical artery development compared to Wnt2-/- animals? For example, Wnt2-/- animal has hypoplastic bladder vs. bladder agenesis in Shh-/-; Wnt2-/- animal develops LUR but Shh-/- does not.

Response: We thank the reviewer for this insightful comment. The model unfortunately implies that *Shh* functions exclusively through *Wnt2,* which in fact is not our intention. We believe, based on our findings, that *Wnt2* is one of the many downstream effectors of *Shh*, which collective control co-development of the bladder and umbilical arteries. Similarly, *Wnt2* expression is likely regulated by additional signals besides *Shh*. To clarify these points, we have revised the model by pointing out additional effectors.

16. The idea that Shh and Wnt signaling affect umbilical artery development is very interesting. Have the authors considered studying whether the umbilical artery or MUR/LUR express Shh or Wnt2 receptors and response genes, as the arrows in the schematic might suggest in Figure 7? If so, what is the spatial and temporal expression pattern during development? This could help elucidate whether the Shh-Wnt2 signaling occurs directly from the cloaca or through some other intermediate tissue or organs.

Response: We appreciate these insights. Indeed, we have considered expression pattern of possible receptors and downstream effectors of *Shh* and *Wnt2*. We have performed spatial transcriptomics of the developing cloaca and are currently in the process of analyzing the spatial data. By examining spatial expression patterns of relevant genes in each of the signaling pathways, future efforts are needed to elucidate the signaling mechanism of co-development of the bladder and the umbilical arteries.

Minor Suggestions:

1. For HREM images in the figures, please consider color-coding the labels to match the color of the corresponding organ to help the reader.

Response: We appreciate the suggestion and updated the figure legends. As suggested, we use a uniform color code for all figures, including green (gut, tail gut, and cloacal epithelium), white (allantoic gut), red (Wolffian duct), yellow (ureteric bud), blue (common nephric duct), aqua (Mullerian duct), and purple (arteries).

2. For all figures, please consider including all abbreviations in the figure legend, not only those new to each figure. For example, mention what Cl and TG stand for in Figure 2 even though they appeared in Figure 1.

Response: We appreciate the suggestion and have updated all the Figure legends to include all abbreviations.

3. Please consider adding explanations of what the arrows in Figure 3A and 3B indicate in within the figure legend.

Response: We appreciate the suggestion. These arrows point to the junction between allantoic and cloaca epithelia. We have updated the figure legend.

4. For Figure S5, please consider annotating key anatomical structures to orient the readers.

Response: To orientated readers, we included directional information, ie. dorsal/ventral (D/V) and rostral/caudal (R/C), major anatomic landmarks such as body stalk (BS), allantois, caudal neural pore, headfold, neural fold, etc. In

© 2026. Published by The Company of Biologists under the terms of the Creative Commons Attribution License (https://creativecommons.org/licenses/by/4.0/).

addition, a video of entire 2D sections of a human embryo was also included, which offers a globe overview.

5. For lines 266-267, please consider adding data and an explanation regarding the significance of Wnt2 in your analysis; for example, clarify that Wnt2 was the top candidate in what particular analysis.

Response: We appreciate the suggestion. *Wnt2* is the top candidates that are differential expressed in the putative bladder mesenchyme compared to the genital tubercle mesenchyme at E11.5. We have included this in the revised manuscript.

6. It would be helpful for the authors to discuss why, in Shh-/- and Wnt2-/- animals, one umbilical artery still develops while the other does not. The reviewer is also curious if it is always one particular side (e.g. right side) that develops in each model.

Response: Thank you for the critical insight. We are unclear why one umbilical artery remains while the other does not. There is no apparent trend of laterality we could observe. The umbilical arteries are essential for embryonic survival. In the case of *Shh* mutants, we speculate that, because of necessity, failure to develop lateral umbilical artery delays the process of degeneration of the medial umbilical artery so that one of them remains. In the case of *Wnt2* mutants, however, it appears that formation of the lateral umbilical arteries is affected while the degeneration process proceeded normally. In addition to molecular determinants, we suspect that hemodynamics may also play an important role thereby contribute to the stochastic variability of the arterial phenotype.

7. It would be helpful for the authors to discuss whether, in addition to cloaca→umbilical artery signaling, there may be umbilical artery→cloaca signaling involved in bladder development.

Response: Thank you for the suggestion. It is indeed possible that co-development of the bladder and umbilical arteries may depend on the bidirectional signals between the umbilical arteries and cloaca. The reciprocal signaling between *Shh* and *Wnt2/2b* has been observed to control co-development of the lung and heart, we suspect that these signals may operate in the similar manner during co-development of the bladder and umbilical arteries, a possibility worthy of future investigation. We have included the discussion in the revised manuscript (Line 366-378).

8. It would be helpful for the authors to mention if developmental defects in bladder has been associated with vascular anomaly in human, if such data is available.

Response: In cases of bladder agenesis birth defects, the affected individuals almost always display severe vascular anomaly consistent with remodeling defect of the umbilical arteries. We have discussed this in the introduction (Line 80-82).

Reviewer 2:

Bladder development remains poorly understood. The dev.205398 describes co-developmental processes of the bladder and umbilical arteries. It also shows such co-development is orchestrated by the outgrowth epithelial signal Shh and mesenchymal signal Wnt2. The MS also contains human embryonic data offering insights for the unexplored topics. The work is compactly summarized and I basically support its publication. I have several points to potentially improve the MS.

Response: We thank the Reviewer for the insights and supports.

1. Wnt2 phenotypes; The Wnt2 deficient bladders are smaller indeed but the phenotypes are not robust. Any possibility of other Wnts involvement?

Response: We appreciate the suggestion. *Wnt2* has a highly conserved and functionally related paralog, *Wnt2b*. *Wnt2* and *Wnt2b* share similar expression pattern during mouse organogenesis, included development of the lung and bladder. Previous reports have shown that these two genes share redundant functions in lung development (Goss AM, et al, Dev Cell 2009; and Peng T, et al., 2013). We do not have access to the *Wnt2/2b* double knockout embryos. However, we do suspect that they

© 2026. Published by The Company of Biologists under the terms of the Creative Commons Attribution License (https://creativecommons.org/licenses/by/4.0/).

share redundant function in bladder development.

2. Shh-Wnt2 signals; Shh coordinates development of both the bladder and umbilical arteries. Wnt2 signaling is restricted to the rostral outgrowth mesenchyme with its expression down-regulated in Shh-null mutants. Is the signal reciprocal in a way of Wnt2 (mesen) to epithelial Shh? In the middle of Fig.7, it in fact shows Shh to Wnt by arrows. But the left Fig shows Wnt2(green) to Shh (red)?

Response: We thank the Reviewer's insight. It is indeed possible that co-development of the bladder and umbilical arteries may depend on the bidirectional signals between the umbilical arteries and cloaca. Given that the reciprocal signaling between *Shh* and *Wnt2/2b* has been observed during development of the cardiopulmonary structures, we suspect that these signals may operate in the similar manner during co-development of the bladder and umbilical arteries. We have updated our model to highlight the possibility.

3. There is such case of reciprocal signal for the co development of the lungs and heart with Wnt2 and Shh. This can be mentioned. https://doi.org/10.1073/pnas.1811624115

Response: We thank the Reviewer for pointing out this relevant publication, which supports our hypothesis that reciprocal signaling between SHH and WNT2/2B regulates co-development of the bladder and umbilical arteries. We have discussed findings of this article and referenced it in the revised manuscript (line 366-373).

Detailed descriptions in Fig 1-3; Detailed data including human embryos are effective to elucidate the coordinated organs development. As for its implications for human birth defects, it has been known that Bladder formation is significantly affected by complications in twin pregnancies with shared placentas and other structural urinary defects can occur compared with singleton. Comments on such based on the current discussions? Such will appeal to wide range of Development readers.

Response: We thank the Reviewer for highlighting this potential connection. We have revised the manuscript to discuss the possibility (line 373-376).

4. Mice are known for pregnancy with multiple litter size. In their data, unlike human embryos, an expected endodermal protrusion was never observed in mouse embryos. However, remodeling of the umbilical arteries is detected in both species. Speculation or Species related comments could be also described?

Response: We thank the Reviewer for this intriguing thought. Our findings imply that umbilical artery remodeling is not associated with litter size. With regard to the differences in endodermal protrusion between human and mouse embryos, it is possible that the allantois-associated extraembryonic visceral endoderm of mouse embryos may have analogous functions as the endodermal protrusion of human embryos despite the anatomic difference. However, this is simply a speculation.

5. Related Shh-Wnt signals around cloaca; About the Wnt-Shh interaction with cloaca and genital tubercle, there is a previous study with Shh (loss) and Wnt (gain of function) mutant analysis. ß-catenin GOF mutation rescues defective GT outgrowth from the cloaca.
https://doi.org/10.1242/dev.039438

Response: We thank the Reviewer for the insight. The Shh-Wnt signaling pathway is indeed critical for development of the genital tubercle. It is possible that spatiotemporal compartmentation of the pathway controls development of different organ system (line 357-360).

6. In sum, the work is effective in elucidating complex coordinated organs development with appropriate revision.

Response: We thank the Reviewer the deep insights and constructive suggestions.

Reviewer 3:

This is a very interesting study focused on the role of the cloaca, the primordium of the bladder, urethra and gut, as an organizing center that promotes normal patterning of umbilical arteries. The authors use mutants lacking Wnt2 and Shh in their studies to elucidate the

© 2026. Published by The Company of Biologists under the terms of the Creative Commons Attribution License (https://creativecommons.org/licenses/by/4.0/).

functions that underlie interactions between the cloaca and Umbilical arteries.

Response: We thank the Reviewer for the deep insights and constructive suggestions.

Comments/Suggestions

1. It would be good to include description of the structure of the Umbilical arteries and their derivation to aid readers in understanding the process.

Response: We appreciate the Reviewer's suggestions. Accordingly, we have included the following language in the revised manuscript (line 205-212):

"The umbilical and fetal circulatory systems form independently. In mice, the umbilical artery originates from mesoderm-derived allantoic angioblasts and first appears around E8.5. At approximately the same time, an independent vessel of confluence (VOC) emerges at the base of the allantois closest to the embryo. It has been proposed that the VOC differentiates into the umbilical artery root that connects directly to the embryonic dorsal aorta. However, remodeling of the umbilical artery roots, particularly its potential relationship to bladder development, has not been reported to date."

2. The visual and textual descriptions of UA formation are very difficult to follow as written. (ie muscle endothelial cells). Related to this, are all of the components derived from the same embryonic primordia? Is there a possible connection between extraembryonic mesoderm and cloacal mesoderm? There have been some studies looking at the derivation of the mesodermal component of the cloaca-it would be good to include some of this data in discussions or in the introduction to evaluate a possible link between cloacal mesoderm and extra-embryonic mesoderm as above.

Response: We thank the Reviewer's suggestions. We have provided a brief description of the developmental process of the UA (see above) to assist readers. We believe that readers will also benefit from the 3D video provided as the supplemental data. As described above, all components of the UA are from the allantois. However, the umbilical artery roots arise independent from VOC of the allantois. While suspected, there is no experimental evidence demonstrating any intermixing between the extraembryonic mesoderm and cloacal mesoderm. The cloacal mesoderm contributes to the development of several structures, including the bladder, genital tubercle, anorectal canal, and the hindlimbs. We have highlighted this in lines 83-86.

3. The embryonic/adult umbilical arteries are well studied, and it is generally accepted that the umbilical artery is derived from extraembryonic mesoderm while the epithelial component of the cloaca is derived from endoderm. As the allantois is not prominent in mice as it is in humans, the issue of cloaca derivation from the allantois is not really present in the mouse field but is discussed mostly in older studies looking at human tissue/fetal malformations. However, one could argue that things are different in humans vs mouse, and that the embryonic origins of the allantois and cloacal tissue are more closely linked in humans vs mice. This issue is not addressed experimentally here, but could be for example by trajectory analysis of human developing embryos using sc RNA-seq for example, or spatial RNA-seq analysis of Paraffin embedded material from human embryos.

Response: We thank the Reviewer the insightful comments. Indeed, the embryonic origins of the allantois and cloacal tissue, particularly the mesodermal components, are poorly characterized. It is possible that they originate from a common progenitor pool. The suggested approaches, *i.e.*, scRNA-seq and/or spatial RNA-seq of human embryos, will help to illuminate this important question in the future.

4. Shh drives an important signaling center in the midline. Its absence leads to many midline-related and laterality abnormalities in the brain, eyes, head/neck skeleton. Shh mutants also exhibit defective cloacal septation and defective bladder differentiation, as well as imperforate anus. One could argue that it is the absence of Shh signaling center is the the major issue, as opposed to 'the cloaca". One could imagine that abnormal tissue size or position for example failure in cloacal septation is going to affect 3-D patterning of structures including the UAs which

© 2026. Published by The Company of Biologists under the terms of the Creative Commons Attribution License (https://creativecommons.org/licenses/by/4.0/).

are closely associated with the bladder, resulting in defects such as single UA. To prove that the cloaca is the source of signals one would have to either identify the cloacal-derived signal (is it Shh or Wnt2?) or evaluate whether UA form when there is cloacal agenesis. Along these lines Is the single umbilical artery phenotype found in individuals without anomalies in cloacal derived structures? This can't really be done from observation of sections or 3D analysis.

Response: We thank the Reviewer the deep insights and comments. Our findings could not differentiate whether cloacal signal(s), such as SHH, may directly and/or indirectly regulate umbilical artery formation. We are in the process to determine whether any of the canonical *Shh* signaling pathway genes are expressed in the umbilical artery mesenchyme, an indication of direct signaling. The single umbilical artery phenotype, in fact, are frequently found in individuals without anomalies in cloacal derived structures. In addition to genetic causes, interference of hemodynamics of the umbilical arteries and others could also result in the phenotype.

4. How does Bmp7 fit in here? Previous studies from other groups suggest that Shh cooperates with Bmp signaling to orchestrate events during cloacal differentiation. The authors should comment on this.

Response: Thank you and we have discussed the synergistic effects between Shh and BMP4/7 in caudal development (line 119-126).

Second decision letter

MS ID#: dev.205398R1

MS TITLE: The cloacal outgrowth orchestrates co-development of the bladder and the umbilical arteries

AUTHORS: Xing Ye, Liguang Xia, Xianfa Yang, Ruirong Tan, Haochuan Zhang, Liming Lei, Jungang Huang, Yingsheng Zhang, Edward Morrisey, Ping Zhu, Zhongrong Li, Naihe Jing and Xue Li

Dear Dr Li,

I have now received all the referees reports on the above manuscript, and have reached a decision. The referees' comments are appended below.

The overall evaluation is positive and we would like to publish a revised manuscript in Development, provided that the referees' comments can be satisfactorily addressed. Please attend to all of the reviewers' comments in your revised manuscript and detail them in your point-by-point response. If you do not agree with any of their criticisms or suggestions explain clearly why this is so. If it would be helpful, you are welcome to contact us to discuss your revision in greater detail. Please send us a point-by-point response indicating your plans for addressing the referees' comments, and we will look over this and provide further guidance.

Reviewer 1

Reviewer comments:
The reviewer appreciate the effort from the authors to address majority of the comments from the reviewer. Minor comments below to strengthen the conclusions of the manuscript:

1. Original major comment 1:
Reviewer 1: In general for HREM studies, it would be helpful to provide more detail on how structures are manually annotated and provide additional data (e.g., H&E and/or lineage-specific immunofluorescence/IHC) to support the accuracy of the manual annotation. More specific instances are mentioned in detailed comments below.

© 2026. Published by The Company of Biologists under the terms of the Creative Commons Attribution License (https://creativecommons.org/licenses/by/4.0/).

Response: We thank the reviewer for this helpful comment. We have included the following description in the revision (line 420-427). Representative 2D images are included in the Supplementary Material to support the accuracy and consistency of the annotation strategy (Figure S1B-D and new Figure S2). To ascertain the annotation, selected structures were confirmed independently using high-resolution serial histological sections (Figure S1_K).

Reviewer 1: Can the authors confirm reference to Figure S2 as mentioned above? Figure S2 as reported in the updated manuscript is referring to Wnt2 RNA in situ hybridization.

2. Original major comment 4:
Reviewer 1: For Figure 2, please consider providing the N and SD for the statistical analysis in panels G and H. Student's t-test were also included.

Response: We updated the figure legend to include the data. Results of Student's t-test were also included.

Reviewer 1: Please can the author indicate on the figure (*) which pairs are statistically significantly different in Figure 2G-H, as suggested in the figure legend?

3. Original major comment 5:
Reviewer 1: The authors suggest that the entire urinary bladder originates from the cloaca without any contribution from the allantois based on serial HREM imaging. It would be helpful to further support this conclusion with lineage tracing studie—either showing that the entire bladder can be lineage-traced back to the cloaca or that the allantois lineage does not contribute to the bladder—to rule out the possibility of cell migration during development.

Response: We thank the reviewer for the insightful comments. As suggested, we used the genetic lineage tracing strategy to indelibly label all the Shh-expression progenitors and their progenies. This strategy enables permeant tagging all the Shh genetic lineages and the tagged cells can be visualized by a highly specific and sensitive X-gal staining protocol. Moreover, the stained embryos were analyzed using HREM 3D approach, which provides a comprehensive overall view as well as single-cell resolution views. Findings from this analysis strongly suggest that the entire bladder can be lineage-traced back to the cloaca (Figure S6, original S5). However, we are unable to trace allantois lineages because no genetic tools are currently available. Therefore, we could not rule out the possibility that the allantois could de-differentiate into the bladder. We have discussed this limitation in the manuscript (line 337-340).

Reviewer 1: Can the authors confirm reference to Figure S6, which the reviewer is not able to identify? At the moment the reviewer is assuming it is referring to the whole embryo X-gal movie (Movie 5). To fully support the conclusion that the entire bladder originate from the cloaca, it would be important to demonstrate that the entire bladder are Shh lineage +. Currently in Movie 5, the X-gal staining shows positivity in the bladder region, but the percentage of X gal+ cells out of all bladder cells remains unclear as it currently stands. It would be helpful to provide a background staining (e.g. H&E) to show the entire bladder stains positive for X-gal. Alternatively, a flow cytometry experiment demonstrating nearly 100% Shh lineage tracing positivity from a bladder sample could be helpful. Also can the authors confirm reference to line 337-340, which in the revised PDF is about Wnt2.

4. Original major comment 8:
Reviewer 1: For lines 230-235, please consider providing additional data such as immunostaining and the number of samples examined to support the conclusion that bladder development is absent. It would be helpful to subsequently interpret the result in the context of findings by Perriton et al. and Haraguchi et al., who found that in Shh-/- mice, the bladder develops although severely hypoplastic.

Response: We thank the reviewer for the insights. We have provided the number of samples examine in the figure legend to support the conclusion. We also addressed the comment of immunostaining below in #9. Regarding the interpretation, we have revised the paragraph and include additional citations as followings:

© 2026. Published by The Company of Biologists under the terms of the Creative Commons Attribution License (https://creativecommons.org/licenses/by/4.0/).

"In addition, the rostral and caudal outgrowth of the UGS was arrested at E12.5, resulting in agenesis of the urinary bladder and genital tubercle as reported previously ,{Haraguchi, 2001 #626;Perriton, 2002 #643;Haraguchi, 2007 #624}. Stage-specific, deletions of Shh further indicate an early phase requirement of Shh signaling cloacal ,septation, formation of the bladder and genital tubercle, and a later phase requirement ,of external genital development {Seifert, 2009 #989;Miyagawa, 2009 #994;Lin, 2009 #3862}. Together, these findings show that epithelial Shh signal is critically required for formation of the bladder and genital tubercle." (line 241-247)

Reviewer 1: The reviewer apologizes if it was not clear in the original comment. In Perriton et al and Haraguchi et al, the findings is the bladder still develops but hypoplastic. This is in contrary to the findings from the authors, which as mentioned above "agenesis of urinary bladder". Can the authors help interpret/explain such difference?

Also could the authors double check text reference? (It is very helpful to the reviewer that the edited text is included in the response, but it current reads from line 252-258 in the PDF)

5. Original major comment 9:
Reviewer 1: For lines 245-246, it would be helpful to provide immunostaining data to show that Shh-lineage and the umbilical artery are mutually exclusive.

Response: We used the genetic lineage tracing strategy to indelibly label all the Shh-expression progenitors and their progenies. This strategy enables permeant tagging all the Shh genetic lineages and the tagged cells can be visualized by a highly specific and sensitive X-gal staining protocol. Moreover, the stained embryos were analyzed using HREM 3D approach, which provides a comprehensive overall view as well as single-cell resolution views. Overall, we believe that these strategies serve as excellent alternatives to immunostaining. We therefore did not perform additional immunostaining.

Reviewer 1: Similar to the comment above, please consider some background staining (H&E or umbilical artery-lineage specific staining)to demonstrate none of the umbilical artery tissue stains positive with X-gal or Shh lineage trace.

6. Original minor comment 4:
Reviewer 1: For Figure S5, please consider annotating key anatomical structures to orient the readers.

Response: To orientated readers, we included directional information, ie.dorsal/ventral (D/V) and rostral/caudal (R/C), major anatomic landmarks such as body stalk (BS), allantois, caudal neural pore, headfold, neural fold, etc. In addition, a video of entire 2D sections of a human embryo was also included, which offers aglobe overview.

Reviewer 1: The MP4 file made available to the reviewer does not seem to contain the update the authors mentioned. Currently it is a video of X-gal staining rotating in different orientations. Would you mind double check please?

<u>Reviewer 2</u>

SUMMARY OF THE ADVANCE MADE IN THIS PAPER AND ITS POTENTIAL SIGNIFICANCE TO THE FIELD

The revised MS responds to the points raised by the reviewer.
Intriguing comments (speculations) on human twins are added.
Descriptions on mouse models of GT outgrowth from cloaca with constitutive active Wnt for Shh-LOF are also added.

The MS will certainly contribute to understanding the coordinated formation of cloaca- bladder and the umbilical arteries.
Furthermore, it will appeal to researchers on human birth defects bridging the fields of developmental biology and medical congenital anomalies.

© 2026. Published by The Company of Biologists under the terms of the Creative Commons Attribution License (https://creativecommons.org/licenses/by/4.0/).

Reviewer 3

SUMMARY OF THE ADVANCE MADE IN THIS PAPER AND ITS POTENTIAL SIGNIFICANCE TO THE FIELD

The manuscript is much improved.

Second revision

Author response to reviewers' comments

I sincerely thank the reviewers for their careful reviews and constructive suggestions, which have clearly improved the ms. I am grateful for the feedback and comments.

One-to-one rebuttal:

Reviewer 1:

a. Original major comment 1:
New Comment: Can the authors confirm reference to Figure S2 as mentioned above? Figure S2 as reported in the updated manuscript is referring to Wnt2 RNA in situ hybridization.
New Response: Figure S2, which is in fact Movie 1, was the original label but was updated to Movie 1 during resubmission. Unfortunately, I did not update all labels during submission, hence the error. I apologize for the confusion.

b. Original major comment 4:
New Comment: Please can the author indicate on the figure (*) which pairs are statistically significantly different in Figure 2G-H, as suggested in the figure legend?
New Response: I have included * in the revised Figure 2G-H.

d. Original major comment 5:
New comment: Can the authors confirm reference to Figure S6, which the reviewer is not able to identify? At the moment the reviewer is assuming it is referring to the whole embryo X-gal movie (Movie 5). To fully support the conclusion that the entire bladder originate from the cloaca, it would be important to demonstrate that the entire bladder are Shh lineage +. Currently in Movie 5, the X-gal staining shows positivity in the bladder region, but the percentage of X gal+ cells out of all bladder cells remains unclear as it currently stands. It would be helpful to provide a background staining (e.g. H&E) to show the entire bladder stains positive for X-gal. Alternatively, a flow cytometry experiment demonstrating nearly 100% Shh lineage tracing positivity from a bladder sample could be helpful. Also can the authors confirm reference to line 337-340, which in the revised PDF is about Wnt2.
New Response: Indeed, Figure S6 refers to Movie 5. As mentioned in Comment #1, this occurs due to formatting error during resubmission.
Our conclusion is based on evidence resulted from analyses of the HREM 3D images, Shh lineage tracing data, and H&E staining of the serial sections. While compelling, we acknowledge that we could not rule out the possibility that the allantois may de-differentiate into the bladder, especially in humans (line 351-352, instead of 337-340. We apology for the error.) A detailed mapping of Shh lineages would provide additional supporting evidence. Nevertheless, it still won't be able to rule out the possibility even if the entire bladder is Shh lineage+. We therefore decided not to pursue this line of investigation.

e. Original major comment 8:
New Comments: The reviewer apologizes if it was not clear in the original comment. In Perriton et al and Haraguchi et al, the findings is the bladder still develops but hypoplastic. This is in contrary to the findings from the authors, which as mentioned above "agenesis of urinary bladder". Can the authors help interpret/explain such difference?
Also could the authors double check text reference? (It is very helpful to the reviewer that the edited text is included in the response, but it current reads from line 252-258 in the PDF)

© 2026. Published by The Company of Biologists under the terms of the Creative Commons Attribution License (https://creativecommons.org/licenses/by/4.0/).

<u>New Response:</u> Based on 3D analysis, we consistently observed that the development is arrested in around E12.5 (line 253). Haraguchi et al. reported similar findings (Figure 7D) and Perriton et al. reported in Figure 5E. While Haraguchi et al. also showed a bladder-like structure in Figure 3G, they did not provide any molecular evidence that the abnormal cyst is in fact the bladder. Given that the urogenital sinus of *Shh*-null mutants is highly distended and deformed, as shown in our 3D images, it is possible that the abnormal cyst is in fact a developmental arrested urogenital sinus visualized on a 2D section. The difference of interpretation likely stems from the difference of experimental strategy – Perriton et al., and Haraguchi et al., are based on 2D histological sections whilst out conclusion is based on 3D images of multiple developmental stages.

Lines 241-247 should have been lines 252-258. We apologize for the error.

f. Original major comment 9:
<u>New Comments:</u> Similar to the comment above, please consider some background staining (H&E or umbilical artery-lineage specific staining) to demonstrate none of the umbilical artery tissue stains positive with X-gal or Shh lineage trace.
<u>New Response:</u> We did consider these options. The umbilical artery-lineage specific staining and/or lineage tracing would be ideal. However, we are currently limited because the ideal molecular tool is currently not available. We did not perform additional H&E staining because 3D HREM already provides the clear evidence.

g. Original minor comment 4:
<u>New Comments:</u> The MP4 file made available to the reviewer does not seem to contain the update the authors mentioned. Currently it is a video of X-gal staining rotating in different orientations. Would you mind double check please?
<u>New Response:</u> We have included a new supplementary figure (Figure S2) with still images of the video to highlight key anatomic structures as recommended.

<u>Third decision letter</u>

MS ID#: dev.205398R2

MS TITLE: The cloacal outgrowth orchestrates co-development of the bladder and the umbilical arteries

AUTHORS: Xing Ye, Liguang Xia, Xianfa Yang, Ruirong Tan, Haochuan Zhang, Liming Lei, Jungang Huang, Yingsheng Zhang, Edward Morrisey, Ping Zhu, Zhongrong Li, Naihe Jing and Xue Li

Dear Dr Li,

I am happy to tell you that your manuscript has been accepted for publication in Development, pending our standard publication integrity checks.

<u>Reviewer 1</u>

The authors have addressed all my previous comments. I have no further recommendations or comments.

© 2026. Published by The Company of Biologists under the terms of the Creative Commons Attribution License (https://creativecommons.org/licenses/by/4.0/).
